# Clinical Outcomes of Hypertonic Saline vs Mannitol Treatment Among Children With Traumatic Brain Injury

**DOI:** 10.1001/jamanetworkopen.2025.0438

**Published:** 2025-03-11

**Authors:** Shu-Ling Chong, Yanan Zhu, Quan Wang, Paula Caporal, Juan D. Roa, Freddy Israel Pantoja Chamorro, Thelma Elvira Teran Miranda, Hongxing Dang, Chin Seng Gan, Qalab Abbas, Ivan J. Ardila, Mohannad Ahmad Antar, Jesús A. Domínguez-Rojas, María Miñambres Rodríguez, Natalia Zita Watzlawik, Natalia Elizabeth Gómez Arriola, Adriana Yock-Corrales, Rubén Eduardo Lasso-Palomino, Ming Mei Xiu, Jacqueline S. M. Ong, Hiroshi Kurosawa, Gabriela Aparicio, Chunfeng Liu, Rujipat Samransamruajkit, Juan C. Jaramillo-Bustamante, Nattachai Anantasit, Yek Kee Chor, Deborah M. Turina, Pei Chuen Lee, Marisol Fonseca Flores, Francisco Javier Pilar Orive, Jane Ng Pei Wen, Sebastián González-Dambrauskas, Jan Hau Lee

**Affiliations:** 1 SingHealth Paediatrics Academic Clinical Programme, Duke-NUS Medical School, Singapore; 2 Department of Emergency Medicine, KK Women’s and Children’s Hospital, Singapore; 3 Singapore Clinical Research Institute, Singapore; 4 Pediatric Intensive Care Unit, Beijing Children’s Hospital, Capital Medical University, Beijing, China; 5 LARed Network Johns Hopkins International Injury Research Unit, Health Systems Program, Department of International Health, Johns Hopkins Bloomberg School of Public Health, Baltimore, Maryland; 6 Pediatric Neurocritical Care, Fundación Homi–Universidad del Bosque, Bogotá, Colombia; 7 Intensive Care Unit, Hospital Infantil Los Ángeles, Universidad de Nariño, Pasto, Colombia; 8 Unidad de Terapia Intensiva Pediátrica, Hospital del Niño Manuel Ascencio Villarroel, Cochabamba, Bolivia; 9 Pediatric Intensive Care Unit, Children’s Hospital of Chongqing Medical University, Chongqing, China; 10 Paediatric Intensive Care Unit, Department of Paediatrics, Faculty of Medicine, University of Malaya, Kuala Lumpur, Malaysia; 11 Department of Pediatrics and Child Health, Aga Khan University, Karachi, Pakistan; 12 Pediatric Critical Care, Clínica Uros, Neiva, Colombia; 13 Pediatric Critical Care Medicine, King Abdullah Specialist Children’s Hospital, National Guard Health Affairs, Riyadh, Saudi Arabia; 14 Department of Pediatrics and Pediatric Critical Care Medicine, Instituto Nacional de Salud del Niño, Lima, Peru; 15 Pediatric Intensive Care Unit, Pediatrics Department, Virgen de la Arrixaca Hospital, Murcia, Spain; 16 Hospital de Pediatría Juan P. Garrahan, Buenos Aires, Argentina; 17 Hospital de Trauma, Asunción, Paraguay; 18 Department of Emergency Medicine, Hospital Nacional de Niños Dr. Carlos Saenz Herrera, Caja Costarricense del Seguro Social, San José, Costa Rica; 19 Fundacion Clínica Valle del Lili, Universidad Icesi, Cali, Colombia; 20 Children’s Hospital of Fudan University, Shanghai, China; 21 Department of Paediatrics, Yong Loo Lin School of Medicine, National University of Singapore, Singapore; 22 Division of Pediatric Critical Care Medicine, Hyogo Prefectural Kobe Children’s Hospital, Kobe, Japan; 23 Pediatric Critical Care Unit, El Hospital Interzonal de Agudos Especializado en Pediatría Sor María Ludovica, Buenos Aires, Argentina; 24 Shengjing Hospital of China Medical University, Shenyang, China; 25 King Chulalongkorn Memorial Hospital, Chulalongkorn University, Bangkok, Thailand; 26 Pediatric Intensive Care Unit, Hospital General de Medellín Luz Castro de Gutiérrez E. S. E. Hospital Pablo Tobón Uribe, Universidad de Antioquia, Medellín, Colombia; 27 LARed Network, Montevideo, Uruguay; 28 Ramathibodi Hospital, Mahidol University, Bangkok, Thailand; 29 Department of Paediatrics, Sarawak General Hospital, Sarawak, Malaysia; 30 Hospital de Niños Ricardo Gutiérrez, Buenos Aires, Argentina; 31 UKM Specialist Children’s Hospital, National University of Malaysia, Kuala Lumpur, Malaysia; 32 Pediatric Critical Care, Mexican Institute of Social Security, Mexico City, Mexico; 33 Pediatric Intensive Care Unit, Pediatric Department, Cruces University Hospital, Biocruces Bizkaia Health Research Institute, Bizkaia, Spain; 34 KK Research Centre, KK Women’s and Children’s Hospital, Singapore; 35 LARed Network, Departamento de Pediatría y Unidad de Cuidados Intensivos de Niños, Centro Hospitalario Pereira Rossell, Facultad de Medicina, Universidad de la República, Montevideo, Uruguay; 36 Children’s Intensive Care Unit, SingHealth Paediatrics Academic Clinical Programme, KK Women’s and Children’s Hospital, Singapore

## Abstract

**Importance:**

The use of hypertonic saline (HTS) vs mannitol in the control of elevated intracranial pressure (ICP) secondary to neurotrauma is debated.

**Objective:**

To compare mortality and functional outcomes of treatment with 3% HTS vs 20% mannitol among children with moderate to severe traumatic brain injury (TBI) at risk of elevated ICP.

**Design, Setting, and Participants:**

This prospective, multicenter cohort study was conducted between June 1, 2018, and December 31, 2022, at 28 participating pediatric intensive care units in the Pediatric Acute and Critical Care Medicine in Asia Network (PACCMAN) and the Red Colaborativa Pediátrica de Latinoamérica (LARed) in Asia, Latin America, and Europe. The study included children (aged <18 years) with moderate to severe TBI (Glasgow Coma Scale [GCS] score ≤13).

**Exposure:**

Treatment with 3% HTS compared with 20% mannitol.

**Main Outcomes and Measures:**

Multiple log-binomial regression analysis was performed for mortality, and multiple linear regression analysis was performed for discharge Pediatric Cerebral Performance Category (PCPC) scores and 3-month Glasgow Outcome Scale–Extended Pediatric Version (GOS-E-Peds) scores. Inverse probability of treatment weighting was also performed using the propensity score method to control for baseline imbalance between groups.

**Results:**

This study included 445 children with a median age of 5.0 (IQR, 2.0-11.0) years. More than half of the patients (279 [62.7%]) were boys, and 344 (77.3%) had severe TBI. Overall, 184 children (41.3%) received 3% HTS, 82 (18.4%) received 20% mannitol, 69 (15.5%) received both agents, and 110 (24.7%) received neither agent. The mortality rate was 7.1% (13 of 184 patients) in the HTS group and 11.0% (9 of 82 patients) in the mannitol group (*P* = .34). After adjusting for age, sex, presence of child abuse, time between injury and hospital arrival, lowest GCS score in the first 24 hours, and presence of extradural hemorrhage, no between-group differences in mortality, hospital discharge PCPC scores, or 3-month GOS-E-Peds scores were observed.

**Conclusions and Relevance:**

In this cohort study of children with moderate to severe TBI, the use of HTS was not associated with increased survival or improved functional outcomes compared with mannitol. Future large multicenter randomized clinical trials are required to validate these findings.

## Key Points

**Question:**

Among children with moderate to severe traumatic brain injury, is the use of 3% hypertonic saline (HTS) associated with better survival and functional outcomes compared with 20% mannitol?

**Findings:**

This cohort study included 445 children treated with 3% HTS, 20% mannitol, both agents, or neither agent. No between-group differences in mortality, discharge Pediatric Cerebral Performance Category Scale scores, or 3-month Glasgow Outcome Scale–Extended Pediatric Version outcomes were observed.

**Meaning:**

These findings suggest that compared with mannitol, HTS was not associated with increased survival or improved functional outcomes.

## Teaser

This cohort study examines clinical outcomes of treatment with 3% hypertonic saline vs 20% mannitol among children with moderate to severe traumatic brain injury (TBI) at risk of increased intracranial pressure.

## Introduction

Traumatic injuries, including traumatic brain injury (TBI), are the leading cause of death and disability among children globally.^1,2^ Children with TBI who develop elevated intracranial pressure (ICP) should receive timely and effective interventions to prevent secondary brain damage.^3^

In the management of elevated ICP in severe TBI, the Brain Trauma Foundation (BTF) recommends the use of 3% hypertonic saline (HTS) as the first-line hyperosmolar therapy.^4,5^ In the largest comparative effectiveness study to date among children with severe TBI and elevated ICP, a bolus dose of 3% HTS outperformed mannitol in observed ICP reductions at ICP greater than 20, 25, and 30 mm Hg.^6^ An earlier randomized clinical trial (RCT) of 30 children given equimolar doses of 3% HTS vs 20% mannitol showed that both were equally effective in the management of elevated ICP.^7^ A systematic review involving 11 studies showed that although both HTS and mannitol lower ICP and improve outcomes in pediatric severe TBI, there were insufficient high-quality data to conclude which of the 2 agents was superior.^8^

Although previous studies have focused on ICP control, there are insufficient data on clinical outcomes comparing patients who receive 3% HTS with those who receive 20% mannitol.^6,7,8^ Therefore, we sought to compare mortality and functional outcomes among children with moderate to severe TBI at risk of elevated ICP who received 3% HTS compared with those who received 20% mannitol. We hypothesized that pediatric patients who received 3% HTS would have comparable mortality and functional outcomes compared with those who received 20% mannitol.

## Methods

We performed this prospective multicenter observational cohort study between June 1, 2018, and December 31, 2022, at participating pediatric intensive care units (PICUs) in the Pediatric Acute and Critical Care Medicine in Asia Network (PACCMAN)^9^ and the Red Colaborativa Pediátrica de Latinoamérica (LARed Network) in Asia, Latin America, and Europe.^10^ LARed sites joined the study in January 2021. We excluded sites without a neurosurgical service. Ethics approval was first obtained in the coordinating country (SingHealth Centralised Institutional Review Board, KK Women’s and Children’s Hospital, Singapore), which required documented informed consent. Subsequently, ethics approval was sought by each individual participating center, and the need for informed consent was determined by local prevailing practices and regulations. The study followed the Strengthening the Reporting of Observational Studies in Epidemiology (STROBE) reporting guideline.

### Participants

We recruited children (aged <18 years) with moderate to severe TBI (Glasgow Coma Scale [GCS] score ≤13) who were admitted to a participating PICU. We excluded children who had a low GCS score from other causes (eg, hypotension and central nervous system infections) that were not related to TBI, as well as children with cardiac arrest after trauma. Children were screened for eligibility upon admission to the PICU.

### Variables

We obtained baseline characteristics, including age, sex, mechanism of injury, time between injury and hospital arrival, presenting and lowest GCS scores in the first 24 hours, and presence of multiple traumas (ie, intrathoracic injuries, intra-abdominal injuries, or long bone fractures). We recorded blood test results on arrival in the PICU, including blood gas, hemoglobin, and sodium levels as well as prothrombin time and international normalized ratio. We also recorded the initial brain computed tomography (CT) findings.

Critical care interventions included mechanical ventilation, use of blood products, inotropes, antiepileptic medications, and ICP and electroencephalogram (EEG) monitoring. Neurosurgical interventions included evacuation of intracranial bleed, decompressive craniectomy, craniotomy, or elevation of depressed skull fracture. We also recorded the opening pressure, daily 6 am pressure values, maximum and minimum ICP, and cerebral perfusion pressure (CPP) values for those who underwent ICP monitoring. We followed each patient’s progress for the presence of seizures (categorized as clinical seizures, subclinical seizures detected on EEG, and subclinical seizures suspected based on vital signs), duration of hospital stay, and PICU length of stay. We also obtained Pediatric Index of Mortality–3 scores to estimate probability of death^11^ on PICU admission.

### Hyperosmolar Therapy

We recorded the use of 3% HTS and 20% mannitol. We included all children who were given both bolus doses and infusions of these medications. We excluded the following outliers: (1) children who received an HTS bolus of more than 10 mL/kg or at an infusion rate of more than 5 mL/kg/h and (2) children who received a 20% mannitol bolus of more than 1.5 g/kg (or the equivalent 7.5 mL/kg).^5^ When describing our study population, we compared those who received 3% HTS alone, 20% mannitol alone, both 3% HTS and 20% mannitol, or neither agent.

### Outcome Variables

Our primary outcomes of interest were mortality and functional outcomes. Mortality was defined as in-hospital mortality. Functional outcomes were measured using the Pediatric Cerebral Performance Category (PCPC) scale at hospital discharge^12,13^ and the Glasgow Outcome Scale–Extended Pediatric Version (GOS-E-Peds) score 3 months after injury. PCPC and GOS-E-Peds scores were documented both as continuous and binary variables. We defined poor functional outcome as a PCPC score of moderate disability, severe disability, vegetative state or coma, or death (PCPC score of 3-6).^14,15^ We defined a poor 3-month GOS-E-Peds score as moderate to severe disability, vegetative state, or death (GOS-E-Peds score of 3-8).^16^ We also recorded secondary outcomes with the Functional Status Scale (FSS) on hospital discharge, with domains including mental status, sensory functioning, communication, motor functioning, feeding, and respiratory status, categorized from normal (score of 1) to very severe dysfunction (score of 5).^11^ A change in FSS score was defined as a change of 3 or greater.^17^ The FSS has been used in previous pediatric trauma cohorts and is reported to be associated with health-related quality of life among children with trauma.^18,19,20,21^

### Data Sources and Measurement

We obtained data from individual medical records. Site investigators uploaded deidentified data to a common REDCap (research electronic data capture) platform (Vanderbilt University) that was hosted by the Singapore Clinical Research Institute.^22^ We did not blind site investigators from the hypothesis. The same persons who reviewed the medical records documented the type of hyperosmolar agent and the clinical outcomes. However, we did ensure that treatment and outcome variables were established a priori with common understanding across sites.

### Statistical Analysis

We describe categorical variables using frequencies and percentages. Continuous variables are described using means (SDs) or medians (IQRs), depending on normality. When comparing between groups, we used the Fisher exact test for categorical variables. Parametric and nonparametric continuous variables were analyzed using the *t* test or the Mann-Whitney *U* for 2-group comparisons and using 1-way analysis of variance (ANOVA) or the Kruskal-Wallis test for multiple group comparisons, respectively. We verified the assumption of equal variances when using 1-way ANOVA. In the descriptive analysis, we present all 4 groups: children who received neither hyperosmolar agent, those who received 3% HTS only, those who received 20% mannitol only, and those who received both agents. Where there were missing data for outcome variables, we present the number of children with complete data in the respective cells in each table (the numbers with complete data were taken as the denominators). Individual and mean ICP and CPP values by day are plotted in scatterplots and line charts, respectively. Linear mixed models for repeated measurements were used to estimate the statistical differences among the 4 groups.

In the analysis on outcomes, we first restricted the analysis to those who received either 3% HTS or 20% mannitol (but not both). Subsequently, we also presented a comparison on clinical outcomes of all 4 groups. We performed multiple log-binomial regression for mortality, adjusting for age, sex, presence of child abuse, time between injury and hospital arrival, lowest GCS score in the first 24 hours, and presence of extradural hemorrhage (EDH). We chose the lowest GCS score in the first 24 hours and not the admission GCS score, given that time between injury and presentation varied greatly between sites and countries due to heterogeneous prehospital trauma systems. Variables that entered the multiple regression were chosen based on previous data, known literature, and univariate significance.^14,23^ We present relative risks (RRs) with corresponding 95% CIs. We also performed multiple linear regression to study the association of hyperosmolar agent with PCPC score on discharge and with 3-month GOS-E-Peds scores. Multicollinearity was checked in multiple regression models by calculating the variance inflation factors (VIFs). We present the adjusted coefficient together with the SE. We considered *P* < .05 (2-tailed) statistically significant.

In addition to adjusting for covariates with multivariable regression, we performed inverse probability of treatment weighting (IPTW) using the propensity score method to control for baseline imbalance between the group who received only 3% HTS and the group who received only 20% mannitol. Propensity scores were derived by fitting a generalized linear model with binomial distribution and logit link with the following baseline characteristic variables: age (continuous), lowest GCS score in the first 24 hours (continuous), blood sodium level (continuous), and presence of child abuse (binary). In cases where the site principal investigators did not enter the lowest GCS score, we imputed with the child’s presenting GCS score. Stabilized IPTW weights were then generated using the propensity score. Baseline covariate balance was assessed using the absolute standardized mean difference (ASMD). An ASMD value of <0.15 indicates negligible imbalance.

We performed subgroup analyses for children with severe TBI (GCS score ≤8) and those who received ICP monitoring. We did not perform multivariable analysis in the latter subgroup because of small numbers. Statistical analysis was performed with R software, version 4.4.0 (R Project for Statistical Computing).

## Results

Twenty-eight PICUs participated in this study (14 in PACCMAN and 14 in LARed). Among the 470 children enrolled, 455 met the eligibility criteria. Ten children were excluded due to 3% HTS dosage outliers, resulting in 445 children for inclusion in this analysis (Figure). A total of 344 patients (77.3%) had severe TBI, and 106 (23.8%) underwent ICP monitoring. A total of 184 children (41.3%) received only 3% HTS, 82 (18.4%) received only 20% mannitol, 69 (15.5%) received both agents, and 110 (24.7%) received neither agent (Figure). The overall median age of our cohort was 5.0 (IQR, 2.0-11.0) years; 279 patients (62.7%) were boys and 166 (37.3%) were girls (Table 1). Abusive head trauma was reported in 27 children (6.1%). Multiple traumas were present in patients who received both agents (51 patients [73.9%]), followed by those who received 20% mannitol (41 [50.0%]), 3% HTS (82 [44.6%]), or neither agent (28 [25.5%]) (overall *P* < .001). The median (IQR) time between injury and hospital arrival was 6.0 (4.0-12.0) hours, 6.0 (3.0-14.8) hours, 4.0 (1.1-9.0) hours, and 2.4 (1.0-9.2) hours for children who received both agents, 20% mannitol, neither agent, and 3% HTS, respectively (overall *P* < .001). The lowest GCS in the first 24 hours was comparable across groups (Table 1).

**Figure.**
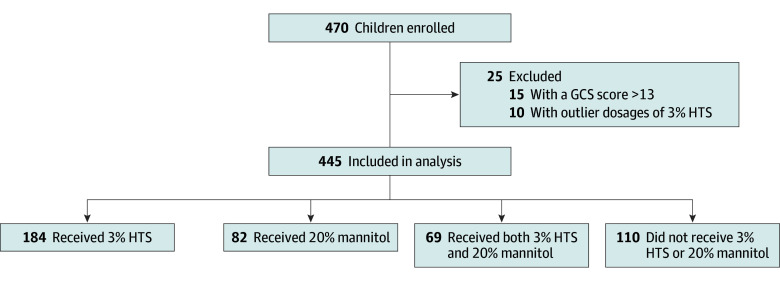
Flow Chart of Patients Analyzed GCS indicates Glasgow Coma Scale; HTS, hypertonic saline.

**Table 1.** Patient Demographics, Injury Characteristics, Blood Investigations, and Imaging^a^

Characteristic	Treatment received (N = 445)				*P* value
	3% HTS (n = 184)	20% Mannitol (n = 82)	Both agents (n = 69)	Neither agent (n = 110)	
Age, median (IQR), y	6.0 (2.0-11.0)	7.0 (3.0-11.8)	5.0 (3.0-9.0)	4.5 (1.0-10.8)	.44^b^
Sex					
Male	123 (66.8)	52 (63.4)	40 (58.0)	64 (58.2)	.39^c^
Female	61 (33.2)	30 (36.6)	29 (42.0)	46 (41.8)	
Mechanism of injury					
Road traffic injury	80 (43.5)	38 (46.3)	37 (53.6)	48 (43.6)	.07^c^
Falls	69 (37.5)	40 (48.8)	25 (36.2)	43 (39.1)	
Child abuse	18 (9.8)	1 (1.2)	2 (2.9)	6 (5.5)	
Others	17 (9.2)	3 (3.7)	5 (7.2)	13 (11.8)	
Time between injury and hospital arrival, median (IQR), h	2.4 (1.0-9.2)	6.0 (3.0-14.8)	6.0 (4.0-12.0)	4.0 (1.1-9.0)	<.001^b^
Lowest GCS score in first 24 h, median (IQR)	6.0 (4.0-8.0)	6.0 (3.0-8.0)	6.0 (5.0-7.0)	7.0 (3.0-10.8)	.21^b^
Presence of multiple traumas	82 (44.6)	41 (50.0)	51 (73.9)	28 (25.5)	<.001^c^
Temperature, mean (SD), °C	36.5 (0.9)	36.5 (1.1)	36.8 (1.1)	36.4 (1.0)	.10^d^
Heart rate, mean (SD), beats/min	117.2 (32.2)	115.4 (30.6)	133.2 (34.9)	113.1 (35.6)	.001^d^
SBP, mean (SD), mm Hg	108.7 (19.2)	108.5 (20.5)	106.8 (18.0)	112.4 (20.2)	.28^d^
Blood test results, median (IQR)					
pH	7.3 (7.2-7.4)	7.3 (7.3-7.4)	7.4 (7.3-7.4)	7.3 (7.3-7.4)	.006^b^
Paco_2_	37.1 (32.9-45.0)	34.4 (29.1-39.1)	33.8 (30.0-39.9)	39.0 (33.7-43.4)	.001^b^
Pao_2_	116.9 (67.5-164.2)	132.0 (86.0-171.0)	156.0 (108.8-193.0)	96.5 (60.2-172.8)	.002^b^
Bicarbonate, mean (SD), mEq/L	19.9 (3.9)	18.5 (3.4)	20.6 (3.6)	19.4 (3.78)	.01^d^
Base excess, mean (SD), mmol/L	−5.6 (5.5)	−7.1 (4.2)	−5.2 (4.4)	−5.9 (5.0)	.12^d^
Hemoglobin, g/dL	10.9 (9.6-12.6)	11.1 (9.1-12.6)	10.5 (8.9-11.8)	11.4 (9.8-12.8)	.17^b^
Sodium, mEq/L	139 (137-141)	140 (138-142)	137 (135-140)	139 (137-141)	<.001^b^
Prothrombin time, s	13.0 (12.1-15.9)	14.7 (13.0-15.9)	14.3 (13.1-15.7)	13.4 (12.8-15.7)	.009^b^
INR	1.2 (1.1-1.3)	1.2 (1.1-1.4)	1.2 (1.1-1.4)	1.1 (1.1-1.2)	.001^b^
CT imaging					
Subarachnoid hemorrhage	43 (23.4)	22 (26.8)	21 (30.4)	19 (17.3)	.21^c^
Subdural hemorrhage	63 (34.2)	19 (23.2)	20 (29.0)	28 (25.5)	.25^c^
Extradural hemorrhage	41 (22.3)	12 (14.6)	18 (26.1)	28 (25.5)	.23^c^
Intraparenchymal or intraventricular bleed	40 (21.7)	30 (36.6)	33 (47.8)	20 (18.2)	<.001^c^
Diffuse axonal injury	17 (9.2)	9 (11.0)	32 (46.4)	6 (5.5)	<.001^c^
Cerebral edema	63 (34.2)	32 (39.0)	45 (65.2)	13 (11.8)	<.001^c^
Presence of midline shift	46 (25.1)	26 (31.7)	11 (15.9)	20 (18.2)	.07^c^
PIM-3 estimated probability of death, median (IQR)	0.05 (0.03-0.07)	0.08 (0.04-0.62)	0.55 (0.05-0.81)	0.05 (0.02-0.09)	<.001^b^

Abbreviations: CT, computed tomography; GCS, Glasgow Coma Scale; HTS, hypertonic saline; INR, international normalized ratio; Paco_2_, partial pressure of carbon dioxide; Pao_2_, partial pressure of oxygen; PIM-3, Pediatric Index of Mortality–3; SBP, systolic blood pressure.

SI conversion factors: To convert bicarbonate to mmol/L, multiply by 1.0; to convert hemoglobin to g/L, multiply by 10.0; to convert sodium to mmol/L, multiply by 1.0.

^a^ Unless indicated otherwise, values are presented as the No. (%) of patients.

^b^ Nonparametric test (Kruskal-Wallis test).

^c^ Fisher exact test.

^d^ Parametric test (1-way analysis of variance).

CT evidence of cerebral edema was present among patients who received both agents (45 [65.2%]), followed by those who received only 20% mannitol (32 [39.0%]), only 3% HTS (63 [34.2%]), or neither agent (13 [11.8%]) (overall *P* < .001) (Table 1). Similarly, among patients who received both agents, nearly half had intraparenchymal or intraventricular bleeds and diffuse axonal injury (33 [47.8%] and 32 [46.4%]), followed by those who received only mannitol (30 [36.6%] and 9 [11.0%]), only HTS (40 [21.7%] and 17 [9.2%]), or neither agent (20 [18.2%] and 6 [5.5%]), respectively (both overall *P* < .001).

Overall, 200 of 445 patients (44.9%) required neurosurgical intervention, including more than half (103 [56.0%]) of the HTS group, followed by those who received neither agent (45 [40.9%]), those who received mannitol (33 [40.2%]), and those who received both agents (19 [27.5%]) (overall *P* < .001) (eTable 1 in Supplement 1). Children who received only HTS had a continuous reduction in daily 6 am ICP values, unlike those who received only mannitol (eFigure 1 and eTable 2 in Supplement 1). Children who received only HTS had mean daily 6 am CPP values on days 1 to 3 that were greater than those for the group who received only mannitol and those for the group who received both agents, but this was not statistically significant (eFigure 2 and eTables 2 and 3 in Supplement 1). Maximum ICP and maximum and minimum CPP readings are reported in eFigures 3 to 5 in Supplement 1. The distribution of dosages for both HTS and mannitol are detailed in eFigures 6 to 8 in Supplement 1.

There was no difference in duration of mechanical ventilation, PICU stay, hospital stay, and occurrence of seizures between children who received HTS compared with those who received mannitol (Table 2). Among 43 of 266 patients (16.2%) with seizures, the majority were detected clinically. Mortality was comparable (13 [7.1%] for the HTS group and 9 [11.0%] for the mannitol group; *P* = .34). Poor PCPC scores on discharge, changes in FSS scores, and poor GOS-E-Peds scores at 3 months were similar between both groups (Table 2).

**Table 2.** Clinical Outcomes After Traumatic Brain Injury

Outcome	Treatment received^a^		*P* value
	3% HTS (n = 184)	20% Mannitol (n = 82)	
Seizures	34/176 (19.3)	9/68 (13.2)	.35^b^
Any seizure			
Clinical seizure	33/34 (97.1)	9/9 (100)	>.99^b^
Subclinical seizure only detected on EEG	1/34 (2.9)	0	
Subclinical seizure suspected based on vital signs	0	0	
Mortality	13/184 (7.1)	9/82 (11.0)	.34^b^
Duration of mechanical ventilation, median (IQR), d	5.0 (2.5-8.0)	4.0 (3.0-6.8)	.69^c^
No. of patients	159	58	NA
Duration of hospital stay, median (IQR), d	16.0 (8.0-29.0)	17.0 (9.5-27.0)	.78^c^
No. of patients	166	71	NA
Duration of PICU stay, median (IQR), d	7.0 (4.0-13.0)	7.0 (4.0-10.0)	.44^c^
No. of patients	181	76	NA
14-d Mechanical ventilation–free days, median (IQR), d	9.0 (4.0-11.0)	9.0 (4.0-11.0)	.81^c^
No. of patients	165	65	NA
28-d Hospital-free days, median (IQR), d	11.0 (0-19.0)	9.0 (0-18.0)	.51^c^
No. of patients	179	80	NA
14-d PICU-free days, median (IQR), d	6.0 (0-10.0)	6.0 (2.0-10.0)	.76^c^
No. of patients	184	80	NA
PCPC score at discharge, median (IQR)	2.0 (1.0-3.0)	2.0 (1.0-2.0)	.67^c^
3-6 (Poor)	57/181 (31.5)	20/81 (24.7)	.31^b^
Increased by ≥2 categories from baseline	42/168 (25.0)	14/75 (18.7)	.32^b^
Final FSS score, median (IQR)	7.0 (6.0-10.0)	7.0 (6.0-9.0)	.37^c^
Change ≥3 (Postinjury score – Preinjury score)	51/181 (28.2)	22/77 (28.6)	>.99^b^
3-mo GOS-E-Peds score, median (IQR)	3.0 (1.0-6.0)	5.0 (1.0-6.0)	.11^c^
3-8 (Poor)	96/180 (53.3)	46/73 (63.0)	.17^b^

Abbreviations: EEG, electroencephalogram; FSS, Functional Status Scale; GOS-E-Peds, Glasgow Outcome Scale–Extended Pediatric Version; HTS, hypertonic saline; NA, not applicable; PCPC, Pediatric Cerebral Performance Category; PICU, pediatric intensive care unit.

^a^ Unless indicated otherwise, values are presented as No./total No. (%) of patients. In the presence of missing data, the No. of patients with complete data are presented in each cell.

^b^ Fisher exact test.

^c^ Nonparametric test (Mann-Whitney *U* test).

In the comparison of all 4 groups (3% HTS only, 20% mannitol only, both agents, and neither agent), children who received both agents had the greatest mortality. In addition, children who received both agents had the longest duration of intubation, hospitalization, and PICU stay and the greatest frequency of poor GOS-E-Peds outcomes compared with those in other groups (eTable 4 in Supplement 1). On performing IPTW adjustment using the propensity score method, the group that received 3% HTS was comparable to the group that received 20% mannitol (eTable 5 in Supplement 1). After adjusting for age, sex, presence of child abuse, time between injury and hospital arrival, lowest GCS score in the first 24 hours, and EDH, there was no difference in mortality between those who received mannitol only compared with those who received HTS only in the multiple log-binomial regression model (adjusted RR, 1.27 [95% CI, 0.58-2.66]; *P* = .52) or the IPTW-adjusted model (adjusted RR, 1.26 [95% CI, 0.56-2.66]; *P* = .56) (Table 3). There was no between-group difference in PCPC scores at discharge in the multiple linear regression model (adjusted coefficient [SE], −0.02 [0.19]; *P* = .91) and the IPTW-adjusted model (adjusted coefficient [SE], −0.10 [0.19]; *P* = .62) (Table 4). For the 3-month GOS-E-Peds scores, we found a marginal (but not statistically significant) increase in the use of mannitol (compared with the use of HTS) and poor outcomes in the multiple linear regression model (adjusted coefficient [SE], 0.56 [0.33]; *P* = .09) and the IPTW-adjusted model (adjusted coefficient [SE], 0.64 [0.33]; *P* = .05) (Table 4).

**Table 3.** Association of Mortality With Hyperosmolar Agent Type and Covariates in Univariate, Multivariable, and IPTW-Adjusted Log-Binomial Regression Analysis^a^

Variable	Univariate model		Multivariable model		IPTW-adjusted model	
	RR (95% CI)	*P* value	RR (95% CI)	*P* value	RR (95% CI)	*P* value
Hyperosmolar therapy						
3% HTS only	1 [Reference]	NA	1 [Reference]	NA	1 [Reference]	NA
20% Mannitol only	1.61 (0.69-3.59)	.25	1.27 (0.58-2.66)	.52	1.26 (0.56-2.66)	.56
Age	0.98 (0.91-1.04)	.43	NA	NA	NA	NA
Sex						
Female	1 [Reference]	NA	1 [Reference]	NA	1 [Reference]	NA
Male	0.66 (0.36-1.23)	.18	NA	NA	NA	NA
Child abuse	1.33 (0.34-3.38)	.62	NA	NA	NA	NA
Time between injury and hospital arrival, h	0.98 (0.94-1.00)	.19	NA	NA	NA	NA
Lowest GCS score in first 24 h	0.56 (0.43-0.68)	<.001	0.50 (0.33-0.67)	<.001	0.49 (0.32-0.67)	<.001
Extradural hemorrhage	0.09 (0.01-0.43)	.02	0.29 (0.02-1.26)	.21	0.28 (0.02-1.21)	.20

Abbreviations: GCS, Glasgow Coma Scale; HTS, hypertonic saline; IPTW, inverse probability treatment weights; NA, not applicable; RR, relative risk.

^a^ Multivariable and IPTW-adjusted RRs (95% CIs) and *P* values are presented for hyperosmolar agents and other variables with univariate significance of <.15.

**Table 4.** Association of Functional Scores With Hyperosmolar Agent Type and Covariates in Univariate, Multivariable, and IPTW-Adjusted Linear Regression Analyses^a^

Variable	Univariate model		Multivariable model		IPTW-adjusted model	
	Coefficient (SE)	*P* value	Adjusted coefficient (SE)	*P* value	Adjusted coefficient (SE)	*P* value
**PCPC score on hospital discharge**						
Hyperosmolar therapy						
3% HTS only	1 [Reference]	NA	1 [Reference]	NA	1 [Reference]	NA
20% Mannitol only	0.001 (0.21)	>.99	−0.02 (0.19)	.91	−0.10 (0.19)	.62
Age	0.001 (0.01)	.93	NA	NA	NA	NA
Sex						
Female	1 [Reference]	NA	1 [Reference]	NA	1 [Reference]	NA
Male	−0.002 (0.15)	.99	NA	NA	NA	NA
Child abuse	0.46 (0.31)	.14	0.45 (0.35)	.19	0.17 (0.36)	.63
Time between injury and hospital arrival, h	<−0.001 (0.004)	.90	NA	NA	NA	NA
Lowest GCS score in first 24 h	−0.20 (0.02)	<.001	−0.20 (0.03)	<.001	−0.20 (0.03)	<.001
Extradural hemorrhage	−0.64 (0.18)	<.001	−0.39 (0.23)	.09	−0.40 (0.23)	.08
**GOS-E-Peds score at 3 mo**						
Hyperosmolar therapy						
3% HTS only	1 [Reference]	NA	1 [Reference]	NA	1 [Reference]	NA
20% Mannitol only	0.61 (0.35)	.09	0.56 (0.33)	.09	0.64 (0.33)	.05
Age	−0.03 (0.03)	.20	NA	NA	NA	NA
Sex						
Female	1 [Reference]	NA	1 [Reference]	NA	1 [Reference]	NA
Male	−0.21 (0.26)	.42	NA	NA	NA	NA
Child abuse	1.25 (0.50)	.01	0.93 (0.57)	.11	1.02 (0.60)	.09
Time between injury and hospital arrival, h	0.001 (0.009)	.95	NA	NA	NA	NA
Lowest GCS score in the first 24 h	−0.27 (0.04)	<.001	−0.23 (0.05)	<.001	−0.23 (0.05)	<.001
Extradural hemorrhage	−1.32 (0.29)	<.001	−1.20 (0.38)	.002	−1.19 (0.38)	.002

Abbreviations: GCS, Glasgow Coma Scale; GOS-E-Peds, Glasgow Outcome Scale–Extended Pediatric Version; HTS, hypertonic saline; IPTW, inverse probability treatment weight; NA, not applicable; PCPC, Pediatric Cerebral Performance Category.

^a^ Multivariable and IPTW-adjusted coefficients (SEs) and *P* values are presented for hyperosmolar agents and other variables with univariate significance of <.15.

In the multivariable linear regression model comparing the 4 groups, children who received both agents had increased mortality risk (adjusted RR, 5.86 [95% CI, 2.40-19.00]; *P* = .001) and poorer GOS-E-Peds scores (adjusted coefficient [SE], 2.14 [0.36]; *P* < .001), with marginal evidence of increased risk for poor PCPC scores at discharge (eTables 6-8 in Supplement 1). In multiple regression models, all VIFs for multicollinearity tests were close to 1, indicating low multicollinearity among the involved variables.

We found consistent results in the subgroup analysis for children with GCS scores of 8 or less (eTables 9-13 in Supplement 1) and those who received ICP monitoring (eTables 14-18 in Supplement 1). There were no statistically significant differences in mortality, discharge PCPC scores, and 3-month GOS-E-Peds scores between those who received HTS vs mannitol.

## Discussion

In this multinational pediatric TBI study, we report the type and dosage of hyperosmolar agents used to treat children with moderate to severe TBI in Asia, Latin America, and Europe. No differences were observed in in-hospital mortality and functional outcomes (using PCPC and FSS scores) between children receiving HTS and those receiving mannitol. Although we observed a marginal increase in the association between use of mannitol (compared to HTS) and poor 3-month GOS-E-Peds scores in the multiple linear regression and IPTW-adjusted models, this finding was not statistically significant.

The BTF recommends ICP monitoring for children with severe TBI and the use of hyperosmolar agents (3% HTS as the drug of choice) for control of elevated ICP.^5^ Use of 3% HTS for elevated ICP is one of the few interventions with a level 2 recommendation.^5^ Prior pediatric studies reported both HTS and mannitol to be useful in elevated ICP, with HTS demonstrating superiority in ICP reductions.^6,7,8^ Our study on moderate to severe TBI showed no difference in mortality, functional outcomes at discharge, duration of mechanical ventilation, PICU stay, or hospital stay between patients treated with 3% HTS and those treated with 20% mannitol. We observed that patients who received HTS had marginally favorable GOS-E-Peds scores at 3 months, but this finding was not statistically significant. The GOS-E-Peds has been reported to offer some advantage over the PCPC scale through the use of a standardized, performance-based instrument that objectively measures cognitive outcomes.^16^ In a systematic review and meta-analysis of 10 RCTs, among which only 1 RCT was conducted with children, there was no evidence that HTS resulted in favorable GOS outcomes at 6 months after injury.^24^ The authors highlighted heterogeneity in GOS reporting and suggested standardizing core TBI outcomes to align future research.^24^

The Approaches and Decisions for Acute Pediatric TBI Trial (ADAPT) investigators reported that HTS was associated with greater ICP reduction for ICP of more than 25 mm Hg after adjustment for confounders, but not at other ICP thresholds, among children with severe TBI.^6^ In our observational study on moderate to severe TBI, we found a steady reduction in daily 6 am ICP values in the group that received HTS, which was not present in the other groups. Although our study population was different from that of the ADAPT investigators and our measurement time points were limited, we found that the use of HTS was associated with gains in ICP reduction compared with mannitol. Future studies should correlate trends on ICP reductions with patient-centric outcomes.

In this study, we found that higher GCS scores were consistently associated with a lower likelihood of mortality and poor functional outcomes. Using the lowest GCS score in the first 24 hours (prior to intervention) and not the presenting GCS score, we recognized the need to allow for evolution of clinical status, given that time between injury and hospital arrival varied between sites and countries. We also observed that the presence of EDH was associated with a lower likelihood of mortality and poor PCPC outcomes. This finding is in keeping with the available literature, in which the outcome and prognosis of children with EDH tends to be excellent when surgical intervention is performed early.^25,26^ We report here that children who received both 3% HTS and 20% mannitol were more likely to experience cerebral edema and diffuse axonal injury. Children who received both agents had an increased mortality risk and poorer GOS-E-Peds scores. These findings suggest that children who received both agents were likely to have been the most severely injured. However, future studies will need to investigate details on clinical severity, the specific dosages of each type of drug, and other TBI management strategies, as well as their association with clinical outcomes.

We reported a child abuse prevalence of 6.1% (27 of 445 children) in this study. This prevalence was lower than that reported by the ADAPT investigators, who described a prevalence of 6.2%, 6.6%, and 5.0% for definite, probable, and possible abuse, respectively.^6^ We attribute the lower prevalence in our study to a less severe TBI cohort and underreporting of child abuse, which has been reported in low- and middle-income countries, especially in Asia.^27,28^ Among children who experienced seizures, most were detected clinically. We recognize that differential resource availability and physician practices could have accounted for the low number of nonclinical seizures that were identified.

### Limitations

This study has some limitations. We observed important differences between our study population and those in other published research. First, important differences exist between the intended patient population for which the BTF guidelines apply and our study cohort. We chose to include not only children with severe TBI but also children with moderate TBI. We found in a previous study that children with moderate TBI are not all similar in clinical phenotype and that children with a GCS score of 9 to 10 had higher rates of neurocritical care utilization and worse functional outcomes compared with those with a GCS score of 11 to 13.^29^ Therefore, we were keen to study children with moderate to severe TBI, not only severe TBI. Second, we found a lower rate of ICP monitoring in participating sites. Unlike the ADAPT investigators who were able to report ICP and CPP data before and after medication administration on all 518 children with severe TBI,^6^ we were unable to do so because the rate of ICP monitoring is much lower and varies widely in our regions, depending on physician practices and resource limitations.^23^ Because treatment of our patient population differed substantially from that described in the ADAPT study and other previous clinical TBI studies, we recognize the need to avoid overgeneralizing our results. However, our findings do highlight how the current recommendations may need to consider settings with different resource availability.

Because this study was observational, we were unable to standardize other treatment goals and could not identify a causal association between the use of HTS (or mannitol) and clinical outcomes. We were unable to investigate whether prehospital standards for TBI management were adhered to.^30^ We also recognize that opening pressure, daily 6 am pressure values, maximum and minimum ICP, and CPP datapoints provide a limited representation of the patients’ actual ICP and CPP. Values were obtained outside of patient interventions (eg, suctioning of endotracheal tube, flushing of arterial line), but could be confounded by patient status (eg, pain and coughing). Future studies should carefully document cumulative doses of hyperosmolar agents because this would provide an indicator of injury severity and PICU practices. Nevertheless, our observational study demonstrates that we were able to collect and synthesize clinical TBI tertiary data across 2 large pediatric intensive care networks. We also obtained very valuable data from regions of the world where trauma disease burden is high and published data on TBI are sparse.

## Conclusions

In this prospective cohort study of pediatric patients with moderate to severe TBI, the use of 3% HTS (compared with 20% mannitol) was not associated with greater survival, reduced hospital length of stay, or better functional scores. Future large multicenter RCTs are required to validate our findings.

## Notes

Supplement 1. **eTable 1.** Medical and Surgical Interventions **eTable 2.** Daily Intracranial Pressure (ICP) and Cerebral Perfusion Pressure (CPP) Readings **eTable 3.** Linear Mixed Model Analysis for Daily Intracranial Pressure (ICP) and Cerebral Perfusion Pressure (CPP) Readings **eTable 4.** Clinical Outcomes Post TBI Among 4 Groups **eTable 5.** Baseline Characteristics of the Study Population Before and After IPTW Adjustment **eTable 6.** Association of Mortality With Hyperosmolar Agent Type and Covariates in Univariate and Multivariable Log-Binomial Regression (4 Groups) **eTable 7.** Association of Pediatric Cerebral Performance Category (PCPC) Score at Discharge With Hyperosmolar Agent Type and Covariates in Univariate and Multivariable Linear Regression (4 Groups) **eTable 8.** Association of Glasgow Outcome Scale–Extended Pediatric Version (GOS-E Peds) Score With Hyperosmolar Agent Type and Covariates in Univariate and Multivariable Linear Regression (4 Groups) **eTable 9.** Subgroup Analysis (Severe TBI With GCS ≤ 8): Patient Demographics, Injury Characteristics, Blood Investigations, and Imaging **eTable 10.** Subgroup Analysis (Severe TBI With GCS ≤ 8): Medical and Surgical Interventions **eTable 11.** Subgroup Analysis (Severe TBI With GCS ≤ 8): Clinical Outcomes Post TBI **eTable 12.** Subgroup Analysis (Severe TBI With GCS ≤ 8): Association of Mortality With Hyperosmolar Agent Type and Covariates in Univariate, Multivariable, and IPTW-Adjusted Log-Binomial Regressions **eTable 13.** Subgroup Analysis (Severe TBI With GCS ≤ 8): Association of Functional Scores With Hyperosmolar Agent Type and Covariates in Univariate, Multivariable, and IPTW-Adjusted Linear Regression **eTable 14.** Subgroup Analysis on Patients Who Received ICP Monitoring: Patient Demographics, Injury Characteristics, Blood Investigations, and Imaging **eTable 15.** Subgroup Analysis on Patients Who Received ICP Monitoring: Medical and Surgical interventions **eTable 16.** Subgroup Analysis on Patients Who Received ICP Monitoring: Clinical Outcomes Post TBI **eTable 17.** Subgroup Analysis on Patients Who Received ICP Monitoring: Association of Mortality With Hyperosmolar Agent Type and Covariates in Univariate Log-Binomial Regression **eTable 18.** Subgroup Analysis on Patients Who Received ICP Monitoring: Association of Functional Scores With Hyperosmolar Agent Type and Covariates in Univariate Linear Regression **eFigure 1.** Individual and Mean of 6 am Intracranial Pressure (ICP) Readings by Day **eFigure 2.** Individual and Mean of 6 am Cerebral Perfusion Pressure (CPP) Readings by Day **eFigure 3.** Individual and Mean of Highest Intracranial Pressure (ICP) Readings by Day **eFigure 4.** Individual and Mean of Highest Cerebral Perfusion Pressure (CPP) Readings by Day **eFigure 5.** Individual and Mean of Lowest Cerebral Perfusion Pressure (CPP) Readings by Day **eFigure 6.** Distribution of Dosages of Hypertonic Saline (HTS) Bolus Loading Dose (mL/kg) **eFigure 7.** Distribution of Dosages of Hypertonic Saline (HTS) Infusion (mL/kg/h) **eFigure 8.** Distribution of Dosages of Mannitol Bolus Loading Dose (mL/kg)

Supplement 2. **Nonauthor Collaborators. **Pediatric Acute and Critical Care Medicine in Asia Network and Red Colaborativa Pediátrica de Latinoamérica

Supplement 3.
Data Sharing Statement
