# Errors in Table

**DOI:** 10.1001/jamanetworkopen.2025.15833

**Published:** 2025-05-13

In the Original Investigation titled “Clinical Outcomes of Hypertonic Saline vs Mannitol Treatment Among Children With Traumatic Brain Injury,”^1^ published March 11, 2025, the data in the last row of Table 1 were incorrect. This article has been corrected.
