# Supplementary material for: Clinical Outcomes of Hypertonic Saline vs Mannitol Treatment Among Children With Traumatic Brain Injury
Source: JAMA Netw Open. 2025 Mar 11;8(3):e250438. doi: 10.1001/jamanetworkopen.2025.0438 (PMC11897838; doi:10.1001/jamanetworkopen.2025.0438)
Supplement: Supplement 1. — eTable 1. Medical and Surgical Interventions eTable 2. Daily Intracranial Pressure (ICP) and Cerebral Perfusion Pressure (CPP) Readings eTable 3. Linear Mixed Model Analysis for Daily Intracranial Pressure (ICP) and Cerebral Perfusion Pressure (CPP) Readings eTable 4. Clinical Outcomes Post TBI Among 4 Groups eTable 5. Baseline Characteristics of the Study Population Before and After IPTW Adjustment eTable 6. Association of Mortality With Hyperosmolar Agent Type and Covariates in Univariate and Multivariable Log-Binomial Regression (4 Groups) eTable 7. Association of Pediatric Cerebral Performance Category (PCPC) Score at Discharge With Hyperosmolar Agent Type and Covariates in Univariate and Multivariable Linear Regression (4 Groups) eTable 8. Association of Glasgow Outcome Scale–Extended Pediatric Version (GOS-E Peds) Score With Hyperosmolar Agent Type and Covariates in Univariate and Multivariable Linear Regression (4 Groups) eTable 9. Subgroup Analysis (Severe TBI With GCS ≤ 8): Patient Demographics, Injury Characteristics, Blood Investigations, and Imaging eTable 10. Subgroup Analysis (Severe TBI With GCS ≤ 8): Medical and Surgical Interventions eTable 11. Subgroup Analysis (Severe TBI With GCS ≤ 8): Clinical Outcomes Post TBI eTable 12. Subgroup Analysis (Severe TBI With GCS ≤ 8): Association of Mortality With Hyperosmolar Agent Type and Covariates in Univariate, Multivariable, and IPTW-Adjusted Log-Binomial Regressions eTable 13. Subgroup Analysis (Severe TBI With GCS ≤ 8): Association of Functional Scores With Hyperosmolar Agent Type and Covariates in Univariate, Multivariable, and IPTW-Adjusted Linear Regression eTable 14. Subgroup Analysis on Patients Who Received ICP Monitoring: Patient Demographics, Injury Characteristics, Blood Investigations, and Imaging eTable 15. Subgroup Analysis on Patients Who Received ICP Monitoring: Medical and Surgical interventions eTable 16. Subgroup Analysis on Patients Who Received ICP Monitoring: Clinical Outcomes Post TBI [file jamanetwopen-e250438-s001.pdf]

Supplemental Online Content

Chong SL, Yanan JZ, Quan W, et al. Clinical outcomes of hypertonic saline vs mannitol treatment among children with traumatic brain injury. *JAMA Netw Open*. 2025;8(3):e250438. doi:10.1001/jamanetworkopen.2025.0438

eTable 1. Medical and Surgical Interventions

eTable 2. Daily Intracranial Pressure (ICP) and Cerebral Perfusion Pressure (CPP) Readings

eTable 3. Linear Mixed Model Analysis for Daily Intracranial Pressure (ICP) and Cerebral Perfusion Pressure (CPP) Readings

eTable 4. Clinical Outcomes Post TBI Among 4 Groups

eTable 5. Baseline Characteristics of the Study Population Before and After IPTW Adjustment

eTable 6. Association of Mortality With Hyperosmolar Agent Type and Covariates in Univariate and Multivariable Log-Binomial Regression (4 Groups)

eTable 7. Association of Pediatric Cerebral Performance Category (PCPC) Score at Discharge With Hyperosmolar Agent Type and Covariates in Univariate and Multivariable Linear Regression (4 Groups)

eTable 8. Association of Glasgow Outcome Scale–Extended Pediatric Version (GOS-E Peds) Score With Hyperosmolar Agent Type and Covariates in Univariate and Multivariable Linear Regression (4 Groups)

eTable 9. Subgroup Analysis (Severe TBI With GCS ≤ 8): Patient Demographics, Injury Characteristics, Blood Investigations, and Imaging

eTable 10. Subgroup Analysis (Severe TBI With GCS ≤ 8): Medical and Surgical Interventions

eTable 11. Subgroup Analysis (Severe TBI With GCS ≤ 8): Clinical Outcomes Post TBI

eTable 12. Subgroup Analysis (Severe TBI With GCS ≤ 8): Association of Mortality With Hyperosmolar Agent Type and Covariates in Univariate, Multivariable, and IPTW-Adjusted Log-Binomial Regressions

eTable 13. Subgroup Analysis (Severe TBI With GCS ≤ 8): Association of Functional Scores With Hyperosmolar Agent Type and Covariates in Univariate, Multivariable, and IPTW-Adjusted Linear Regression

eTable 14. Subgroup Analysis on Patients Who Received ICP Monitoring: Patient Demographics, Injury Characteristics, Blood Investigations, and Imaging

eTable 15. Subgroup Analysis on Patients Who Received ICP Monitoring: Medical and Surgical interventions

eTable 16. Subgroup Analysis on Patients Who Received ICP Monitoring: Clinical Outcomes Post TBI

eTable 17. Subgroup Analysis on Patients Who Received ICP Monitoring: Association of Mortality With Hyperosmolar Agent Type and Covariates in Univariate Log-Binomial Regression

© 2025 Chong SL et al. *JAMA Network Open*.

eTable 18. Subgroup Analysis on Patients Who Received ICP Monitoring: Association of Functional Scores With Hyperosmolar Agent Type and Covariates in Univariate Linear Regression

eFigure 1. Individual and Mean of 6 AM Intracranial Pressure (ICP) Readings by Day

eFigure 2. Individual and Mean of 6 AM Cerebral Perfusion Pressure (CPP) Readings by Day

eFigure 3. Individual and Mean of Highest Intracranial Pressure (ICP) Readings by Day

eFigure 4. Individual and Mean of Highest Cerebral Perfusion Pressure (CPP) Readings by Day

eFigure 5. Individual and Mean of Lowest Cerebral Perfusion Pressure (CPP) Readings by Day

eFigure 6. Distribution of Dosages of Hypertonic Saline (HTS) Bolus Loading Dose (mL/kg)

eFigure 7. Distribution of Dosages of Hypertonic Saline (HTS) Infusion (mL/kg/h)

eFigure 8. Distribution of Dosages of Mannitol Bolus Loading Dose (mL/kg)

This supplemental material has been provided by the authors to give readers additional information about their work.

© 2025 Chong SL et al. *JAMA Network Open*.

eTable 1. Medical and Surgical Interventions

**Patients, No. (%)**	**Patients who received Hypertonic Saline (N=184)**	**Patients who received 20% Mannitol (N=82)**	**Patients who received both agents (N=69)**	**Patients who did not receive any hyperosmolar therapy (N=110)**	**p value**
Medical management					
Intubated (within 24 hours)	158 (85.9)	69 (85.2)	53 (76.8)	76 (69.1)	.003
Use of anti-epileptic medications	146 (79.8)	61 (74.4)	43 (62.3)	75 (68.8)	.03[a]
Electroencephalogram monitoring	44 (23.9)	12 (14.6)	13 (18.8)	12 (10.9)	.03[a]
Inotrope use	95 (51.6)	28 (34.1)	18 (26.1)	22 (20.0)	<.001[a]
Use of blood products (overall)	80 (43.7)	45 (54.9)	32 (46.4)	40 (36.4)	.08[a]
Use of red blood cells	72 (39.1)	41 (50.0)	25 (36.2)	37 (33.6)	.13[a]
Use of fresh frozen plasma	24 (13.0)	19 (23.2)	20 (29.0)	8 (7.3)	<.001[a]
Barbiturates	23 (12.5)	15 (18.3)	6 (8.7)	10 (9.1)	.20[a]
Sedative Medications	147 (79.9)	49 (59.8)	48 (69.6)	50 (45.5)	<.001[a]
Paralytic medications (not including RSI medications for intubation)	47 (25.5)	18 (22.0)	7 (10.1)	14 (12.7)	.008
Temperature control (within 24 hours)	6 (3.3)	2 (2.4)	5 (7.2)	0 (0.0)	.05
Neurosurgical intervention					
Any neurosurgical intervention	103 (56.0)	33 (40.2)	19 (27.5)	45 (40.9)	<.001[a]
ICP monitoring	62 (33.7)	9 (11.0)	16 (23.2)	19 (17.3)	<.001[a]
Decompressive craniectomy	30 (16.3)	9 (11.0)	7 (10.1)	6 (5.5)	.04[a]
Evacuation of bleed	44 (23.9)	21 (25.6)	10 (14.5)	27 (24.5)	.34[a]

a. Fisher's Exact test
b. Non-parametric test (Kruskal Wallis test)

RSI: Rapid sequence intubation; IQR: Interquartile range; ICP: Intracranial pressure

© 2025 Chong SL et al. *JAMA Network Open*.

eTable 2. Daily Intracranial Pressure (ICP) and Cerebral Perfusion Pressure (CPP) Readings

Agent(s) used	Mean			Standard Deviation (SD)		
	**Day 1**	**Day 2**	**Day 3**	**Day 1**	**Day 2**	**Day 3**
**Maximum Intracranial Pressure (ICP) (mmHg)**						
Hypertonic saline only	24.9	23.0	22.2	10.7	9.1	9.2
Mannitol only	26.6	26.8	18.5	9.8	9.7	6.5
Both	23.4	24.3	22.4	7.8	9.4	9.2
None	19.4	20.7	21.7	6.4	6.1	10.1
**6am Intracranial Pressure (ICP) (mmHg)**						
Hypertonic saline only	16.6	14.9	13.5	8.0	7.6	6.7
Mannitol only	13.9	18.5	12.5	5.7	10.5	4.7
Both	18.4	18.4	15.4	9.2	6.6	4.9
None	17.1	15.9	17.2	5.3	5.0	9.0
**Maximum Cerebral Perfusion Pressure (CPP) (mmHg)**						
Hypertonic saline only	73.4	74.1	74.5	14.6	15.7	14.4
Mannitol only	57.1	73.1	67.9	20.5	12.2	16.2
Both	66.6	66.3	67.2	20.3	17.1	15.6
None	73.5	69.0	70.9	15.9	18.5	14.9
**Minimum Cerebral Perfusion Pressure (CPP) (mmHg)**						
Hypertonic saline only	44.4	46.4	50.5	10.0	12.2	11.1
Mannitol only	39.7	47.0	52.8	15.6	10.0	6.6
Both	51.2	49.9	51.5	19.6	19.7	18.5
None	43.0	42.9	43.4	10.4	11.0	11.4
**6am Cerebral Perfusion Pressure (CPP) (mmHg)**						
Hypertonic saline only	58.1	59.1	61.5	10.2	13.9	14.2
Mannitol only	53.0	56.4	59.1	16.4	14.3	9.8
Both	57.3	55.6	57.9	17.9	18.8	16.7
None	54.8	57.8	57.3	12.2	17.3	14.9

© 2025 Chong SL et al. *JAMA Network Open*.

eTable 3. Linear Mixed Model Analysis for Daily Intracranial Pressure (ICP) and Cerebral Perfusion Pressure (CPP) Readings

**Maximum Intracranial Pressure (ICP)**		
	Coefficient (SE)	*P* value
Hyperosmolar therapy -none	reference	reference
Hyperosmolar therapy -Hypertonic saline only	3.39 (2.17)	0.12
Hyperosmolar therapy -Mannitol only	2.69 (3.17)	0.40
Hyperosmolar therapy -Both	3.72 (2.71)	0.17
**6am Intracranial Pressure (ICP)**		
	Coefficient (SE)	*P* value
Hyperosmolar therapy -none	reference	reference
Hyperosmolar therapy -Hypertonic saline only	0.04 (1.85)	0.98
Hyperosmolar therapy -Mannitol only	-1.10 (2.69)	0.68
Hyperosmolar therapy -Both	2.10 (2.30)	0.36
**Maximum Cerebral Perfusion Pressure (CPP)**		
	Coefficient (SE)	*P* value
Hyperosmolar therapy -none	reference	reference
Hyperosmolar therapy -Hypertonic saline only	0.82 (3.67)	0.82
Hyperosmolar therapy -Mannitol only	-6.84 (5.68)	0.23
Hyperosmolar therapy -Both	-4.18 (4.67)	0.37
**Minimum Cerebral Perfusion Pressure (CPP)**		
	Coefficient (SE)	*P* value
Hyperosmolar therapy -none	reference	reference
Hyperosmolar therapy -Hypertonic saline only	2.83 (2.35)	0.23
Hyperosmolar therapy -Mannitol only	0.81 (3.63)	0.82
Hyperosmolar therapy -Both	6.15 (2.99)	0.04
**6am Cerebral Perfusion Pressure (CPP)**		
	Coefficient (SE)	*P* value
Hyperosmolar therapy -none	reference	reference
Hyperosmolar therapy -Hypertonic saline only	1.42 (2.94)	0.63
Hyperosmolar therapy -Mannitol only	-2.78 (4.60)	0.55
Hyperosmolar therapy -Both	-0.01 (3.74)	0.99

© 2025 Chong SL et al. *JAMA Network Open*.

eTable 4. Clinical Outcomes Post TBI Among 4 Groups

Patients, No. (%)	Patients who received Hypertonic Saline (N=184)	Patients who received 20% Mannitol (N=82)	Patients who received both agents (N=69)	Patients who did not receive any hyperosmolar therapy (N=110)	P value
Seizures	34/176 (19.3)	9/68 (13.2)	8/66 (12.1)	29/95 (30.5)	.01[a]
Any seizure<br>- Clinical seizures<br>- Subclinical seizures only detect on EEG<br>- Subclinical seizures suspected based on vital signs	 33/34 (97.1)<br>1/34 (2.9)<br>0 (0.0)	 9/9 (100.0)<br>0/9 (0.0)<br>0/9 (0.0)	 8/8 (100.0)<br>0/8 (0.0)<br>0/8 (0.0)	 26/29 (89.7)<br>3/29 (10.3)<br>0/29 (0.0)	.57[a]
Mortality	13 (7.1)	9 (11.0)	12 (17.4)	4 (3.6)	.01[a]
Duration of mechanical ventilation, median (IQR), days	N=159<br>5.00<br>[2.50, 8.00]	N=58<br>4.00<br>[3.00, 6.75]	N=44<br>6.00<br>[3.00, 9.00]	N=78<br>3.00<br>[1.00, 6.00]	.006[b]
Duration of hospital stay, median (IQR), days	N=166<br>16.00<br>[8.00, 29.00]	N=71<br>17.00<br>[9.50, 27.00]	N=57<br>17.00<br>[11.00, 28.00]	N=104<br>11.00<br>[6.00, 20.25]	.01[b]
Duration of ICU stay, median (IQR), days	N=181<br>7.00<br>[4.00, 13.00]	N=76<br>7.00<br>[4.00, 10.00]	N=68<br>11.00<br>[5.00, 17.00]	N=108<br>4.00<br>[2.00, 7.25]	<.001[b]
14-day mechanical ventilation free days, median (IQR), days	N=165<br>9.00<br>[4.00, 11.00]	N=65<br>9.00<br>[4.00, 11.00]	N=56<br>7.00<br>[0.00, 10.00]	N=79<br>10.00<br>[8.00, 13.00]	<.001[b]
28–day hospital free days, median (IQR), days	N=179<br>11.00 [0.00, 19.00]	N=80<br>9.00<br>[0.00, 18.00]	7.00<br>[0.00, 16.00]	N=108<br>16.00 [5.00, 21.25]	.001[b]
14-day ICU free days, median (IQR), days	6.00 [0.00, 10.00]	N=80<br>6.00<br>[2.00, 10.00]	0.00<br>[0.00, 5.00]	N=109<br>10.00<br>[6.00, 12.00]	<.001[b]
Poor PCPC (Defined as 3-6)	57/181 (31.5)	20/81 (24.7)	21 (30.4)	25/107 (23.4)	.42 [a]
Poor PCPC (Defined as increase in PCPC by 2 or more categories from the baseline)	42/168 (25.0)	14/75 (18.7)	9/57 (15.8)	21/103 (20.4)	.46[a]
Final Functional Status Score (FSS), median (IQR)	N=181<br>7.00<br>[6.00, 10.00]	N=77<br>7.00<br>[6.00, 9.00]	N=67<br>9.00<br>[6.00, 11.00]	N=109<br>6.00<br>[6.00, 9.00]	.07[b]
FSS-post – FSS-pre >=3	51/181 (28.2)	22/77 (28.6)	34/67 (50.7)	27/109 (24.8)	.003[a]
3-month GOS-E Peds Score, median (IQR)	N=180<br>3.00<br>[1.00, 6.00]	N=73<br>5.00<br>[1.00, 6.00]	5.00<br>[4.00, 7.00]	N=97<br>1.00<br>[1.00, 5.00]	<.001[b]
Poor GOS-E Score (3-8)	96/180 (53.3)	46/73 (63.0)	56 (81.2)	35/97 (36.1)	<.001[a]

a. Fisher's Exact test
b. Non-parametric test (Kruskal Wallis test)

© 2025 Chong SL et al. *JAMA Network Open*.

* P <0.05

EEG: Electroencephalogram; IQR: Interquartile range; ICU: Intensive care unit; PCPC: Pediatric Cerebral Performance Category; FSS: Functional status scale; GOS-E Peds: Glasgow Outcome Score-Extended Pediatrics

In the presence of missing data, we presented the N=Number of complete data in each cell respectively.

© 2025 Chong SL et al. *JAMA Network Open*.

eTable 5. Baseline Characteristics of the Study Population Before and After IPTW Adjustment

			**Before IPTW adjustment**	**After IPTW adjustment**
**Patients, No. (%)**	**Patients who received Hypertonic Saline (N=184)**	**Patients who received 20% Mannitol (N=82)**	**ASMD**	**ASMD**
Age, median (IQR), years	6.00 [2.00, 11.00]	7.00 [3.00, 11.8]	0.161	0.04
Child Abuse	18 (9.8)	1 (1.2)	0.386	0.12
Lowest GCS in first 24 hours, median (IQR)	6 [4, 8]	6 [3, 8]	0.056	0.04
Sodium, median (IQR), mmol/L	139.00 [137.00, 141.00]	140.05 [137.85, 142.07]	0.265	0.07

IPTW: in verse probability treatment weights; ASMD: absolute standardized mean difference; GCS: Glasgow Coma Scale; IQR: Interquartile range; Na: Sodium levels

© 2025 Chong SL et al. *JAMA Network Open*.

eTable 6. Association of Mortality With Hyperosmolar Agent Type and Covariates in Univariate and Multivariable Log-Binomial Regression (4 Groups)

	Univariate model		Multivariable model	
	RR (95% CI)	*P* value	RR (95% CI)	*P* value
Hyperosmolar therapy - none	reference	-	reference	-
Hyperosmolar therapy - Hypertonic saline only	1.94 (0.71, 6.77)	.24	2.17 (0.83, 7.30)	.15
Hyperosmolar therapy - Mannitol only	3.02 (1.02, 10.83)	.06	2.76 (1.00, 9.45)	.07
Hyperosmolar therapy - Both	4.78 (1.75, 16.60)	.005	5.86 (2.40, 19.00)	.001
Age	0.98 (0.91, 1.04)	.43	-	-
Sex (Male)	0.66 (0.36, 1.23)	.18	-	-
Child abuse	1.33 (0.34, 3.38)	.62	-	-
Time between injury and arrival (hours)	0.98 (0.94, 1.00)	.19	-	-
Lowest GCS in first 24 hours	0.56 (0.43, 0.68)	<.001	0.55 (0.43, 0.67)	<.001
Extradural haemorrhage	0.09 (0.01, 0.43)	.02	-.14 (0.008, 0.58)	.04

RR: risk ratio; GCS: Glasgow Coma Scale
Multivariable RR (95%CI) and p values were presented for hyperosmolar agents and other variables with univariate significance of <0.15.

© 2025 Chong SL et al. *JAMA Network Open*.

eTable 7. Association of Pediatric Cerebral Performance Category (PCPC) Score at Discharge With Hyperosmolar Agent Type and Covariates in Univariate and Multivariable Linear Regression (4 Groups)

	Univariate model		Multivariable model	
	Coefficient (SE)	*P* value	Coefficient (SE)	*P* value
Hyperosmolar therapy -none	reference	-	reference	-
Hyperosmolar therapy -Hypertonic saline only	0.32 (0.19)	.09	0.15 (0.18)	.39
Hyperosmolar therapy -Mannitol only	0.32 (0.23)	.16	0.12 (0.21)	.59
Hyperosmolar therapy -Both	0.60 (0.24)	.01	0.42 (0.22)	.06
Age	0.001 (0.01)	.93	-	-
Sex (Male)	-0.002 (0.15)	.99	-	-
Child abuse	0.46 (0.31)	.14	0.21 (0.29)	.46
Time between injury and arrival (hours)	-0.0005 (0.004)	.90	-	-
Lowest GCS in first 24 hours	-0.20 (0.02)	<.001	-0.18 (0.02)	<.001
Extradural haemorrhage	-0.64 (0.18)	<.001	-0.35 (0.17)	.04

SE: standard error; GCS: Glasgow Coma Scale
Multivariable coefficient (SE) and p values were presented for hyperosmolar agents and other variables with univariate significance of <0.15.

© 2025 Chong SL et al. *JAMA Network Open*.

eTable 8. Association of Glasgow Outcome Scale–Extended Pediatric Version (GOS-E Peds) Score With Hyperosmolar Agent Type and Covariates in Univariate and Multivariable Linear Regression (4 Groups)

	Unadjusted coefficient (SE)	P value	Adjusted coefficient (SE)	P value
Hyperosmolar therapy - none	reference	-	Reference	-
Hyperosmolar therapy - Hypertonic saline only	0.82 (0.31)	.008	0.57 (0.29)	.05
Hyperosmolar therapy - Mannitol only	1.43 (0.38)	<.001	1.17 (0.36)	.001
Hyperosmolar therapy - Both	2.33 (0.38)	<.001	2.14 (0.36)	<.001
Age	-0.03 (0.03)	.20	-	-
Sex (Male)	-0.21 (0.26)	.42	-	-
Child abuse	1.25 (0.50)	.01	1.09 (0.46)	.02
Time between injury and arrival (hours)	0.001 (0.009)	.95	-	-
Lowest GCS/presenting GCS	-0.27 (0.04)	<.001	-0.21 (0.04)	<.001
Extradural haemorrhage	-1.32 (0.29)	<.001	-0.90 (0.28)	.001

SE: standard error; GCS: Glasgow Coma Scale
Multivariable coefficient (SE) and p values were presented for hyperosmolar agents and other variables with univariate significance of <0.15.

© 2025 Chong SL et al. *JAMA Network Open*.

eTable 9. Subgroup Analysis (Severe TBI With GCS ≤ 8): Patient Demographics, Injury Characteristics, Blood Investigations, and Imaging

**Patients, No. (%)**	**Patients who received Hypertonic Saline (N=146)**	**Patients who received 20% Mannitol (N=67)**	**Patients who received both agents (N=60)**	**Patients who did not receive any hyperosmolar therapy (N=71)**	**p value**
Age, median (IQR), years	5.00 [2.00, 11.00]	7.00 [3.00, 11.50]	5.00 [2.75, 9.00]	4.00 [1.00, 10.00]	.11[a]
Male sex<br>Female sex	97 (66.4)<br>49 (33.6)	45 (67.2)<br>22 (32.8)	34 (56.7)<br>26 (43.3)	43 (60.6)<br>28 (39.4)	.49[b]
Mechanism of injury<br>Road traffic injury<br>Falls<br>Child abuse<br>Others	 65 (44.5)<br>51 (34.9)<br>16 (11.0)<br>14 (9.6)	 32 (47.8)<br>32 (47.8)<br>1 (1.5)<br>2 (3.0)	 35 (58.3)<br>19 (31.7)<br>2 (3.3)<br>4 (6.7)	 28 (39.4)<br>27 (38.0)<br>5 (7.0)<br>11 (15.5)	.03[b]
Time between injury and arrival, median (IQR), hours	2.00 [1.00, 7.50]	5.00 [3.00, 12.50]	6.00 [3.69, 9.25]	6.00 [1.58, 9.00]	<.001[a]
Lowest GCS in first 24 hours, median (IQR)	6.00 [3.00, 7.00]	6.00 [3.00, 6.50]	6.00 [4.00, 7.00]	5.00 [3.00, 7.00]	.37[a]
Presence of multiple trauma	69 (47.3)	35 (52.2)	46 (76.7)	24 (33.8)	<.001[b]
Temperature, mean (SD), °C	36.40 (0.93)	36.51 (1.15)	36.78 (1.15)	36.45 (1.22)	.15[c]
Heart rate, mean (SD), per min	121.14 (34.53)	116.16 (30.82)	133.93 (35.72)	117.62 (35.89)	.03[c]
Systolic BP, mean (SD), mmHg	107.45 (21.64)	108.90 (21.90)	106.38 (18.63)	110.33 (22.04)	.74[c]
Blood investigations					
pH, median (IQR)	7.32 [7.24, 7.38]	7.34 [7.25, 7.40]	7.37 [7.33, 7.42]	7.30 [7.23, 7.37]	.01[a]
PaCO2, median (IQR)	37.00 [32.20, 45.00]	33.50 [28.85, 38.65]	33.75 [30.30, 39.55]	37.90 [33.00, 44.80]	.008[a]
PaO2, median (IQR)	120.00 [80.00, 169.80]	151.40 [85.93, 179.25]	163.10 [115.40, 194.00]	127.00 [68.50, 191.50]	.06[a]
Bicarbonate, mean (SD), mmol/L	19.79 (3.81)	18.37 (3.30)	20.48 (3.60)	18.52 (3.91)	.003[c]
Base excess, mean (SD), mmol/L	-5.91 (5.33)	-7.37 (4.24)	-5.51 (4.43)	-7.05 (5.25)	.10[c]
Hemoglobin, median (IQR), g/dL	10.80 [9.60, 12.40]	11.05 [9.62, 12.57]	10.80 [9.07, 12.22]	10.10 [9.45, 12.40]	.83[a]
Sodium, median (IQR), mmol/L	139.00 [137.00, 141.00]	140.10 [138.00, 142.70]	137.45 [135.07, 140.12]	139.00 [137.00, 142.00]	.002[a]
Prothrombin Time, median (IQR), sec	13.45 [12.20, 16.35]	14.80 [13.25, 16.00]	14.30 [13.35, 15.85]	13.40 [12.80, 17.10]	.07[a]

© 2025 Chong SL et al. *JAMA Network Open*.

Patients, No. (%)	Patients who received Hypertonic Saline (N=146)	Patients who received 20% Mannitol (N=67)	Patients who received both agents (N=60)	Patients who did not receive any hyperosmolar therapy (N=71)	p value
International normalized ratio, median (IQR)	1.19 [1.09, 1.33]	1.24 [1.11, 1.36]	1.23 [1.14, 1.38]	1.12 [1.01, 1.25]	.01[a]
Computed Tomography					
Subarachnoid hemorrhage	36 (24.7)	19 (28.4)	18 (30.0)	15 (21.4)	.66[b]
Subdural hemorrhage	52 (35.6)	17 (25.4)	16 (26.7)	19 (27.1)	.35[b]
Extradural hemorrhage	28 (19.2)	9 (13.4)	14 (23.3)	10 (14.3)	.42[b]
Intraparenchymal or intraventricular bleed	31 (21.2)	27 (40.3)	32 (53.3)	15 (21.4)	<.001[b]
Diffuse axonal injury	16 (11.0)	9 (13.4)	32 (53.3)	6 (8.6)	<.001[b]
Cerebral edema	54 (37.0)	28 (41.8)	42 (70.0)	12 (17.1)	<.001[b]
Presence of midline shift	33 (22.8)	24 (35.8)	7 (11.7)	12 (17.1)	.01[b]
PIM-3 predicted probability of death, median (IQR)	1.00 [0.99, 1.00]	1.00 [1.00, 1.00]	1.00 [1.00, 1.00]	1.00 [0.99, 1.00]	.02[a]

a. Non-parametric test (Kruskal Wallis test)
b. Fisher's Exact test
c. Parametric test (1 way ANOVA)

IQR: Interquartile range; SD: Standard deviation; PIM-3: pediatric index of mortality-3

© 2025 Chong SL et al. *JAMA Network Open*.

eTable 10. Subgroup Analysis (Severe TBI With GCS ≤ 8): Medical and Surgical Interventions

Patients, No. (%)	Patients who received Hypertonic Saline (N=146)	Patients who received 20% Mannitol (N=67)	Patients who received both agents (N=60)	Patients who did not receive any hyperosmolar therapy (N=71)	p value
Medical management					
Intubated (within 24 hours)	141 (96.6)	62 (93.9)	51 (85.0)	65 (91.5)	.03
Use of anti-epileptic medications	120 (82.8)	52 (77.6)	38 (63.3)	58 (82.9)	.02[a]
Electroencephalogram monitoring	40 (27.4)	10 (14.9)	13 (21.7)	9 (12.7)	.04[a]
Inotrope use	83 (56.8)	26 (38.8)	17 (28.3)	20 (28.2)	<.001[a]
Use of blood products (overall)	70 (48.3)	37 (55.2)	29 (48.3)	34 (47.9)	.78[a]
Use of red blood cells	62 (42.5)	34 (50.7)	23 (38.3)	31 (43.7)	.54[a]
Use of fresh frozen plasma	21 (14.4)	16 (23.9)	19 (31.7)	8 (11.3)	.007[a]
Barbiturates	21 (14.4)	13 (19.4)	4 (6.7)	10 (14.1)	.23[a]
Sedative Medications	132 (90.4)	43 (64.2)	42 (70.0)	42 (59.2)	<.001[a]
Paralytic medications (not including RSI medications for intubation)	40 (27.4)	16 (23.9)	6 (10.0)	11 (15.5)	.02
Temperature control (within 24 hours)	5 (3.4)	2 (3.0)	4 (6.7)	0 (0.0)	.20
Neurosurgical intervention					
Any neurosurgical intervention	85 (58.2)	25 (37.3)	15 (25.0)	29 (40.8)	<.001[a]
ICP monitoring	54 (37.0)	6 (9.0)	13 (21.7)	16 (22.5)	<.001[a]
Decompressive craniectomy	27 (18.5)	7 (10.4)	5 (8.3)	5 (7.0)	.05[a]
Evacuation of bleed	32 (21.9)	16 (23.9)	7 (11.7)	16 (22.5)	.30[a]

a. Fisher’s Exact test
b. Non-parametric test (Kruskal Wallis test)

RSI: Rapid sequence intubation; IQR: Interquartile range; ICP: Intracranial pressure

© 2025 Chong SL et al. *JAMA Network Open*.

eTable 11. Subgroup Analysis (Severe TBI With GCS ≤ 8): Clinical Outcomes Post TBI

**Patients, No. (%)**	**Patients who received Hypertonic Saline (N=146)**	**Patients who received 20% Mannitol (N=67)**	**P value**
Seizures	32/139 (23.0)	8/55 (14.5)	.24[a]
Any seizure<br>- Clinical seizures<br>- Subclinical seizures only detect on EEG<br>- Subclinical seizures suspected based on vital signs	 31/32 (96.9)<br>1/32 (3.1)<br>0 (0.0)	 8/8 (100.0)<br>0 (0.0)<br>0 (0.0)	1.00[a]
Mortality	13 (8.9)	9 (13.4)	.34[a]
Duration of mechanical ventilation, median (IQR), days	N=137<br>5.00 [3.00, 9.00]	N=50<br>4.00 [3.00, 6.75]	.50[b]
Duration of hospital stay, median (IQR), days	N=129<br>17.00 [10.00, 33.00]	N=57<br>18.00 [11.00, 27.00]	.78[b]
Duration of ICU stay, median (IQR), days	N=143<br>8.00 [4.00, 13.00]	N=62<br>7.00 [4.00, 10.00]	.16[b]
14-day mechanical ventilation free days, median (IQR), days	N=143<br>8.00 [4.00, 11.00]	N=57<br>9.00 [3.00, 11.00]	.97[b]
28–day hospital free days, median (IQR), days	N=142<br>8.00 [0.00, 17.00]	N=66<br>8.00 [0.00, 16.00]	.92[b]
14-day ICU free days, median (IQR), days	5.00 [0.00, 9.00]	N=66<br>6.00 [1.25, 9.00]	.39[b]
PCPC at discharge, median (IQR)	N=144<br>2.00 [1.00, 3.00]	N=66<br>2.00 [1.00, 3.00]	.63[b]
Poor PCPC (Defined as 3-6)	54/144 (37.5)	20/66 (30.3)	.35[a]
Poor PCPC (Defined as increase in PCPC by 2 or more categories from the baseline)	39/131 (29.8)	14/60 (23.3)	.39[a]
Final Functional Status Score (FSS), median (IQR)	N=144<br>7.00 [6.00, 10.00]	N=63<br>7.00 [6.00, 9.50]	.34[b]
FSS-post – FSS-pre >=3	46/144 (31.9)	20/63 (31.7)	1.00[a]
3-month GOS-E Peds Score, median (IQR)	N=143<br>5.00 [1.00, 6.00]	N=60<br>5.00 [1.00, 7.00]	.18[b]
Poor GOS-E Score (3-8)	87/143 (60.8)	40/60 (66.7)	.53[a]

a. Fisher's Exact test
b. Non-parametric test (Mann-Whitney U test)

EEG: Electroencephalogram; IQR: Interquartile range; ICU: Intensive care unit; PCPC: Pediatric Cerebral Performance Category; FSS: Functional status scale; GOS-E Peds: Glasgow Outcome Score-Extended Pediatrics
In the presence of missing data, we presented the N=Number of complete data in each cell respectively.

© 2025 Chong SL et al. *JAMA Network Open*.

eTable 12. Subgroup Analysis (Severe TBI With GCS ≤ 8): Association of Mortality With Hyperosmolar Agent Type and Covariates in Univariate, Multivariable, and IPTW-Adjusted Log-Binomial Regressions

	Univariate model		Multivariable model		IPTW-adjusted model	
	RR (95% CI)	*P* value	RR (95% CI)	*P* value	RR (95% CI)	*P* value
Hyperosmolar therapy - Hypertonic saline only	reference	-	reference	-	reference	-
Hyperosmolar therapy - Mannitol only	1.51 (0.65, 3.32)	.31	1.27 (0.58, 2.65)	.53	1.26 (0.56, 2.65)	.55
Age	0.98 (0.92, 1.04)	.57	-	-	-	-
Sex (Male)	0.63 (0.35, 1.16)	.14	1.27 (0.59, 3.25)	.58	1.38 (0.62, 3.80)	.48
Child abuse	1.14 (0.29, 2.88)	.81	-	-	-	-
Time between injury and arrival (hours)	0.98 (0.94, 1.01)	.28	-	-	-	-
Lowest GCS in first 24 hours	0.57 (0.44, 0.71)	<.001	0.51 (0.33, 0.69)	<.001	0.50 (0.32, 0.68)	<.001
Extradural hemorrhage	0.13 (0.01, 0.56)	.04	0.28 (0.02, 1.23)	.20	0.23 (0.01,1.18)	.18

SE: standard error.
IPTW: inverse probability treatment weights; RR: risk ratio; GCS: Glasgow Coma Scale
Multivariable and IPTW-adjusted RR (95% CI) and p values were presented for hyperosmolar agents and other variables with univariate significance of <0.15

© 2025 Chong SL et al. *JAMA Network Open*.

eTable 13. Subgroup Analysis (Severe TBI With GCS ≤ 8): Association of Functional Scores With Hyperosmolar Agent Type and Covariates in Univariate, Multivariable, and IPTW-Adjusted Linear Regression

	Univariate model		Multivariable model		IPTW-adjusted model	
**Pediatric Cerebral Performance Category (PCPC) score on hospital discharge**						
	Coefficient (SE)	*P* value	Adjusted coefficient (SE)	*P* value	Adjusted coefficient (SE)	*P* value
Hyperosmolar therapy -Hypertonic saline only	reference	-	reference	-	reference	-
Hyperosmolar therapy -Mannitol only	-0.002 (0.24)	.99	-0.08 (0.23)	.72	-0.13 (0.23)	.56
Age	0.009 (0.02)	.61	-	-	-	-
Sex (Male)	0.02 (0.19)	.93	-	-	-	-
Child abuse	0.17 (0.35)	.63	-	-	-	-
Time between injury and arrival (hours)	0.002 (0.005)	.71	-	-	-	-
Lowest GCS in first 24 hours	-0.28 (0.05)	<.001	-0.29 (0.06)	<.001	-0.29 (0.06)	<.001
Extradural hemorrhage	-0.55 (0.24)	.02	-0.52 (0.29)	.07	-0.54 (0.29)	.06
**Glasgow Outcome Scale- Extended Peds (GOS-E Peds) score at 3-months**						
Hyperosmolar therapy -Hypertonic saline only	reference	-	reference	-	reference	-
Hyperosmolar therapy -Mannitol only	0.52 (0.39)	.19	0.48 (0.38)	.22	0.50 (0.38)	.18
Age	-0.02 (0.03)	.53	-	-	-	-
Sex (Male)	-0.27 (0.30)	.36	-	-	-	-
Child abuse	0.90 (0.54)	.10	0.83 (0.63)	.19	0.91 (0.66)	.17
Time between injury and arrival (hours)	0.01 (0.01)	.24	-	-	-	-
Lowest GCS in the first 24 hours	-0.24 (0.08)	.002	-0.24 (0.09)	.01	-0.26 (0.09)	.006
Extradural hemorrhage	-1.24 (0.37)	.001	-1.58 (0.46)	<.001	-1.67 (0.46)	<.001

SE: standard error; IPTW: inverse probability treatment weights; GCS: Glasgow Coma Scale; Multivariable and IPTW-adjusted coefficient (SE) and p values were presented for hyperosmolar agents and other variables with univariate significance of <0.15

eTable 14. Subgroup Analysis on Patients Who Received ICP Monitoring: Patient Demographics, Injury Characteristics, Blood Investigations, and Imaging

Patients, No. (%)	Patients who received Hypertonic Saline (N=62)	Patients who received 20% Mannitol (N=9)	Patients who received both agents (N=16)	Patients who did not receive any hyperosmolar therapy (N=19)	p value
Age, median (IQR), years	5.00 [2.00, 9.75]	7.00 [2.00, 9.00]	4.50 [2.67, 10.50]	1.00 [0.62, 6.50]	.15[a]
Male sex<br>Female sex	41 (66.1)<br>21 (33.9)	4 (44.4)<br>5 (55.6)	11 (68.8)<br>5 (31.2)	12 (63.2)<br>7 (36.8)	.62[b]
Mechanism of injury<br>Road traffic injury<br>Falls<br>Child abuse<br>Others	 29 (46.8)<br>16 (25.8)<br>9 (14.5)<br>8 (12.9)	 4 (44.4)<br>5 (55.6)<br>0 (0.0)<br>0 (0.0)	 7 (43.8)<br>9 (56.2)<br>0 (0.0)<br>0 (0.0)	 6 (31.6)<br>7 (36.8)<br>2 (10.5)<br>4 (21.1)	.14[b]
Time between injury and arrival, median (IQR), hours	2.50 [1.00, 7.75]	6.00 [0.75, 8.00]	6.50 [3.64, 11.75]	3.92 [0.94, 9.00]	.14[a]
Lowest GCS in first 24 hours, median (IQR)	6.00 [3.00, 7.00]	7.00 [6.00, 9.00]	7.00 [5.00, 8.00]	5.00 [3.00, 7.50]	.10[a]
Presence of multiple trauma	32 (51.6)	5 (55.6)	9 (56.2)	7 (36.8)	.63[b]
Temperature, mean (SD), °C	36.36 (1.09)	36.59 (0.31)	36.89 (1.02)	36.03 (1.51)	.16[c]
Heart rate, mean (SD), per min	119.88 (33.55)	117.62 (18.02)	132.07 (35.93)	115.06 (26.75)	.49[c]
Systolic BP, mean (SD), mmHg	110.54 (20.49)	109.88 (16.19)	110.14 (21.75)	110.67 (25.54)	1.00[c]
Blood investigations					
pH, median (IQR)	7.32 [7.25, 7.39]	7.35 [7.31, 7.35]	7.40 [7.36, 7.42]	7.28 [7.15, 7.37]	.02[a]
PaCO2, median (IQR)	40.75 [34.00, 45.00]	35.60 [33.50, 44.00]	32.50 [29.12, 34.45]	41.00 [35.30, 49.75]	.02[a]
PaO2, median (IQR)	117.40 [61.00, 156.00]	114.00 [100.00, 226.00]	152.50 [109.12, 204.70]	73.80 [56.38, 207.50]	.19[a]
FiO2, median (IQR)	50.00 [30.00, 52.50]	42.50 [33.75, 62.50]	40.00 [21.00, 40.00]	50.00 [26.25, 90.00]	.12[a]
Bicarbonate, mean (SD), mmol/L	20.26 (2.95)	19.41 (1.58)	20.32 (2.51)	18.91 (3.90)	.35[c]
Base excess, mean (SD), mmol/L	-5.51 (4.73)	-6.68 (1.45)	-5.49 (2.40)	-6.32 (4.55)	.80[c]
Hemoglobin, median (IQR), g/dL	11.00 [9.30, 12.60]	9.30 [8.60, 10.50]	10.15 [8.70, 11.45]	10.10 [9.20, 11.70]	.46[a]
Sodium, median (IQR), mmol/L	139.00 [136.00, 141.00]	138.00 [137.40, 141.00]	137.00 [134.75, 138.18]	140.00 [138.00, 141.00]	.03[a]

© 2025 Chong SL et al. *JAMA Network Open*.

Patients, No. (%)	Patients who received Hypertonic Saline (N=62)	Patients who received 20% Mannitol (N=9)	Patients who received both agents (N=16)	Patients who did not receive any hyperosmolar therapy (N=19)	p value
Prothrombin Time, median (IQR), sec	14.60 [12.93, 18.95]	14.80 [14.20, 15.70]	13.90 [13.35, 14.82]	13.35 [12.50, 18.08]	.82[a]
International normalized ratio, median (IQR)	1.20 [1.08, 1.50]	1.28 [1.19, 1.29]	1.22 [1.16, 1.30]	1.20 [1.06, 1.37]	.82[a]
Computed Tomography					
Subarachnoid hemorrhage	25 (40.3)	5 (55.6)	8 (50.0)	5 (26.3)	.39[b]
Subdural hemorrhage	28 (45.2)	4 (44.4)	5 (31.2)	7 (36.8)	.79[b]
Extradural hemorrhage	14 (22.6)	1 (11.1)	7 (43.8)	5 (26.3)	.30[b]
Intraparenchymal or intraventricular bleed	16 (25.8)	2 (22.2)	5 (31.2)	5 (26.3)	.98[b]
Diffuse axonal injury	4 (6.5)	1 (11.1)	6 (37.5)	1 (5.3)	.009[b]
Cerebral edema	29 (46.8)	3 (33.3)	8 (50.0)	4 (21.1)	.19[b]
Presence of midline shift	15 (24.2)	3 (33.3)	6 (37.5)	5 (26.3)	.70[b]
PIM-3 predicted probability of death, median (IQR)	1.00 [1.00, 1.00]	1.00 [1.00, 1.00]	1.00 [1.00, 1.00]	1.00 [1.00, 1.00]	.17[a]

a. Non-parametric test (Kruskal Wallis test)
b. Fisher's Exact test
c. Parametric test (1 way ANOVA)

IQR: Interquartile range; SD: Standard deviation; PIM-3: pediatric index of mortality-3

© 2025 Chong SL et al. *JAMA Network Open*.

eTable 15. Subgroup Analysis on Patients Who Received ICP Monitoring: Medical and Surgical interventions

**Patients, No. (%)**	**Patients who received Hypertonic Saline (N=62)**	**Patients who received 20% Mannitol (N=9)**	**Patients who received both agents (N=16)**	**Patients who did not receive any hyperosmolar therapy (N=19)**	**p value**
Medical management					
Intubated (within 24 hours)	58 (93.5)	9 (100.0)	13 (81.2)	18 (94.7)	.28[a]
Use of anti-epileptic medications	55 (88.7)	8 (88.9)	9 (56.2)	17 (89.5)	.01[a]
Electroencephalogram monitoring	24 (38.7)	3 (33.3)	4 (25.0)	6 (31.6)	.76[a]
Inotrope use	39 (62.9)	4 (44.4)	3 (18.8)	9 (47.4)	.02[a]
Use of blood products (overall)	35 (56.5)	7 (77.8)	11 (68.8)	13 (68.4)	.49[a]
Use of red blood cells	33 (53.2)	7 (77.8)	8 (50.0)	12 (63.2)	.47[a]
Use of fresh frozen plasma	11 (17.7)	2 (22.2)	6 (37.5)	4 (21.1)	.40[a]
Barbiturates	14 (22.6)	2 (22.2)	6 (37.5)	2 (10.5)	.31[a]
Sedative Medications	60 (96.8)	7 (77.8)	15 (93.8)	17 (89.5)	.16[a]
Paralytic medications (not including RSI medications for intubation)	22 (35.5)	5 (55.6)	4 (25.0)	4 (21.1)	.27[a]
Temperature control (within 24 hours)	3 (4.8)	0 (0.0)	4 (25.0)	0 (0.0)	.01[a]
Neurosurgical intervention					
Any neurosurgical intervention	62 (100.0)	9 (100.0)	16 (100.0)	19 (100.0)	NA
ICP monitoring	62 (100.0)	9 (100.0)	16 (100.0)	19 (100.0)	NA
Decompressive craniectomy	16 (25.8)	3 (33.3)	5 (31.2)	1 (5.3)	.20[a]
Evacuation of bleed	17 (27.4)	1 (11.1)	6 (37.5)	5 (26.3)	.57[a]

a. Fisher's Exact test
b. Non-parametric test (Kruskal Wallis test)

RSI: Rapid sequence intubation; IQR: Interquartile range; ICP: Intracranial pressure

© 2025 Chong SL et al. *JAMA Network Open*.

eTable 16. Subgroup Analysis on Patients Who Received ICP Monitoring: Clinical Outcomes Post TBI

**Patients, No. (%)**	**Patients who received Hypertonic Saline (N=62)**	**Patients who received 20% Mannitol (N=9)**	**P value**
Seizures	12/58 (20.7)	2 (22.2)	1.00[a]
Any seizure<br>- Clinical seizures<br>- Subclinical seizures only detect on EEG<br>- Subclinical seizures suspected based on vital signs	 1112 (91.7)<br>1/12 (8.3)<br>0 (0.0)	 2/2 (100.0)<br>0 (0.0)<br>0 (0.0)	1.00[a]
Mortality	7 (11.3)	0 (0.0)	.58[a]
Duration of mechanical ventilation, median (IQR), days	N=57<br>7.00 [5.00, 11.00]	7.00 [6.00, 8.00]	.87[b]
Duration of hospital stay, median (IQR), days	N=52<br>25.50 [16.50, 41.75]	23.00 [21.00, 29.00]	.75[b]
Duration of ICU stay, median (IQR), days	N=60<br>12.00 [6.00, 18.25]	10.00 [10.00, 12.00]	.97[b]
14-day mechanical ventilation free days, median (IQR), days	6.00 [0.25, 9.00]	7.00 [6.00, 8.00]	.66[b]
28–day hospital free days, median (IQR), days	N=59<br>0.00 [0.00, 10.00]	5.00 [0.00, 7.00]	.61[b]
14-day ICU free days, median (IQR), days	0.00 [0.00, 4.75]	4.00 [2.00, 4.00]	.26[b]
PCPC at discharge, median (IQR)	N=60<br>2.00 [1.75, 4.00]	2.00 [1.00, 2.00]	.047[b]
Poor PCPC (Defined as 3-6)	27/60 (45.0)	1 (11.1)	.07[a]
Poor PCPC (Defined as increase in PCPC by 2 or more categories from the baseline)	19/53 (35.8)	1 (11.1)	.25[a]
Final Functional Status Score (FSS), median (IQR)	N=60<br>8.00 [6.00, 11.00]	7.00 [6.00, 10.00]	.62[b]
FSS-post – FSS-pre >=3	23/60 (38.3)	3 (33.3)	1.00[a]
3-month GOS-E Peds Score, median (IQR)	5.00 [2.25, 6.00]	N=8<br>4.00 [1.75, 5.00]	.22[b]
Poor GOS-E Score (3-8)	46 (74.2)	5/8 (62.5)	.67[a]

c. Fisher's Exact test
d. Non-parametric test (Mann-Whitney U test)

EEG: Electroencephalogram; IQR: Interquartile range; ICU: Intensive care unit; PCPC: Pediatric Cerebral Performance Category; FSS: Functional status scale; GOS-E Peds: Glasgow Outcome Score-Extended Pediatrics
In the presence of missing data, we presented the N=Number of complete data in each cell respectively.

© 2025 Chong SL et al. *JAMA Network Open*.

eTable 17. Subgroup Analysis on Patients Who Received ICP Monitoring: Association of Mortality With Hyperosmolar Agent Type and Covariates in Univariate Log-Binomial Regression

	Univariate model	
	RR (95% CI)	*P* value
Hyperosmolar therapy - Hypertonic saline only	reference	-
Hyperosmolar therapy - Mannitol only	$6.79e^{-8}$ (NA, $8.83e^{+80}$)	.99
Age	0.93 (0.77, 1.07)	.35
Sex (Male)	0.93 (0.24, 4.34)	.92
Child abuse	2.88 (0.46, 10.90)	.16
Time between injury and arrival (hours)	0.99 (0.95, 1.02)	.77
Lowest GCS in first 24 hours	0.54 (0.27, 0.82)	.02
Extradural hemorrhage	0.42 (0.02, 2.20)	.41

© 2025 Chong SL et al. *JAMA Network Open*.

eTable 18. Subgroup Analysis on Patients Who Received ICP Monitoring: Association of Functional Scores With Hyperosmolar Agent Type and Covariates in Univariate Linear Regression

	Univariate model			
	Pediatric Cerebral Performance Category (PCPC) score on hospital discharge		Glasgow Outcome Scale-Extended Peds (GOS-E Peds) score at 3-months	
	Coefficient (SE)	*P* value	Coefficient (SE)	*P* value
Hyperosmolar therapy -Hypertonic saline only	reference	-	reference	-
Hyperosmolar therapy -Mannitol only	-1.12 (0.55)	.05	0.96 (0.88)	.28
Age	-0.04 (0.03)	.15	-0.03 (0.05)	.51
Sex (Male)	0.32 (0.31)	.30	-0.41 (0.47)	.39
Child abuse	1.25 (0.46)	.01	1.21 (0.73)	.10
Time between injury and arrival (hours)	-0.005 (0.01)	.70	0.01 (0.02)	.56
Lowest GCS in first 24 hours	-0.21 (0.06)	<.001	-0.33 (0.08)	<.001
Extradural hemorrhage	-0.14 (0.34)	.69	-0.68 (0.51)	.19

© 2025 Chong SL et al. *JAMA Network Open*.

eFigure 1. Individual and Mean of 6 AM Intracranial Pressure (ICP) Readings by Day

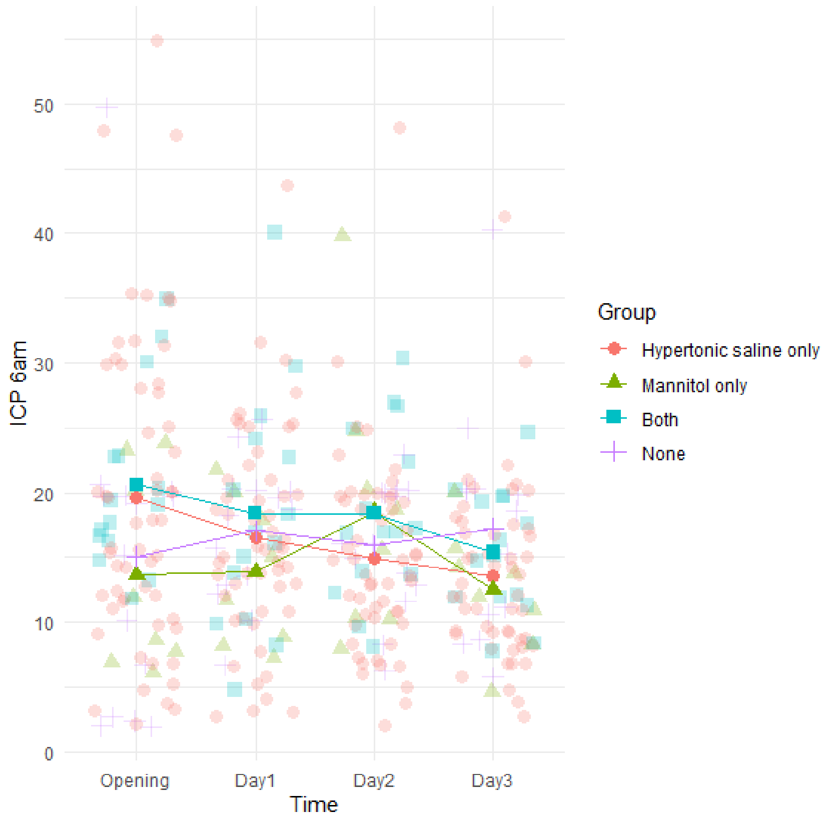

© 2025 Chong SL et al. *JAMA Network Open*.

eFigure 2. Individual and Mean of 6 AM Cerebral Perfusion Pressure (CPP) Readings by Day

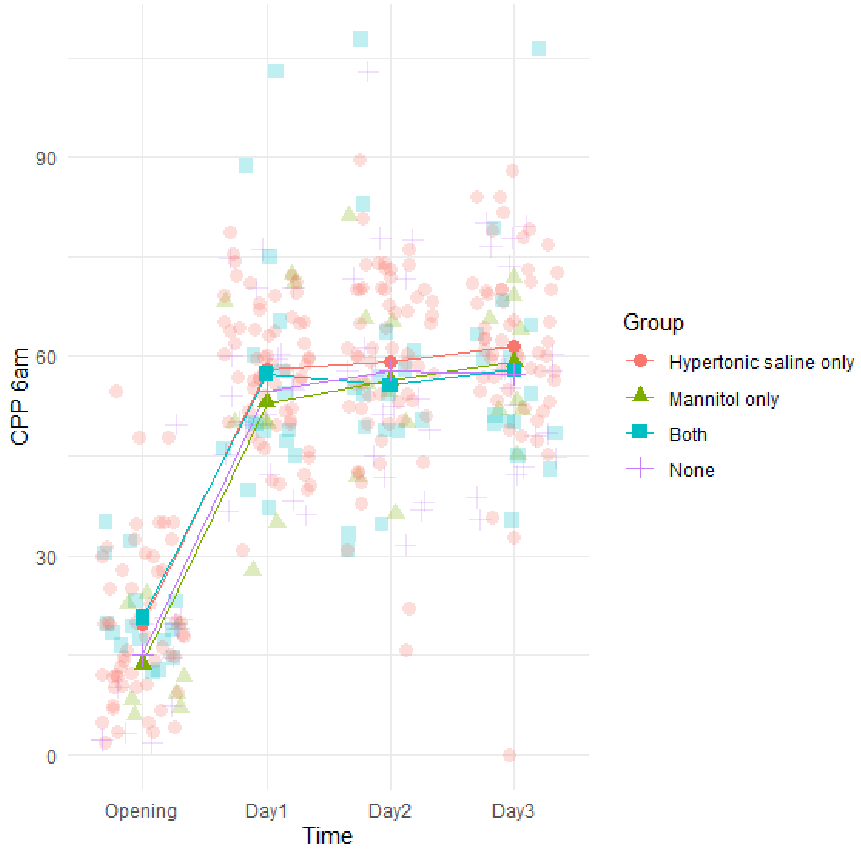

© 2025 Chong SL et al. *JAMA Network Open*.

eFigure 3. Individual and Mean of Highest Intracranial Pressure (ICP) Readings by Day

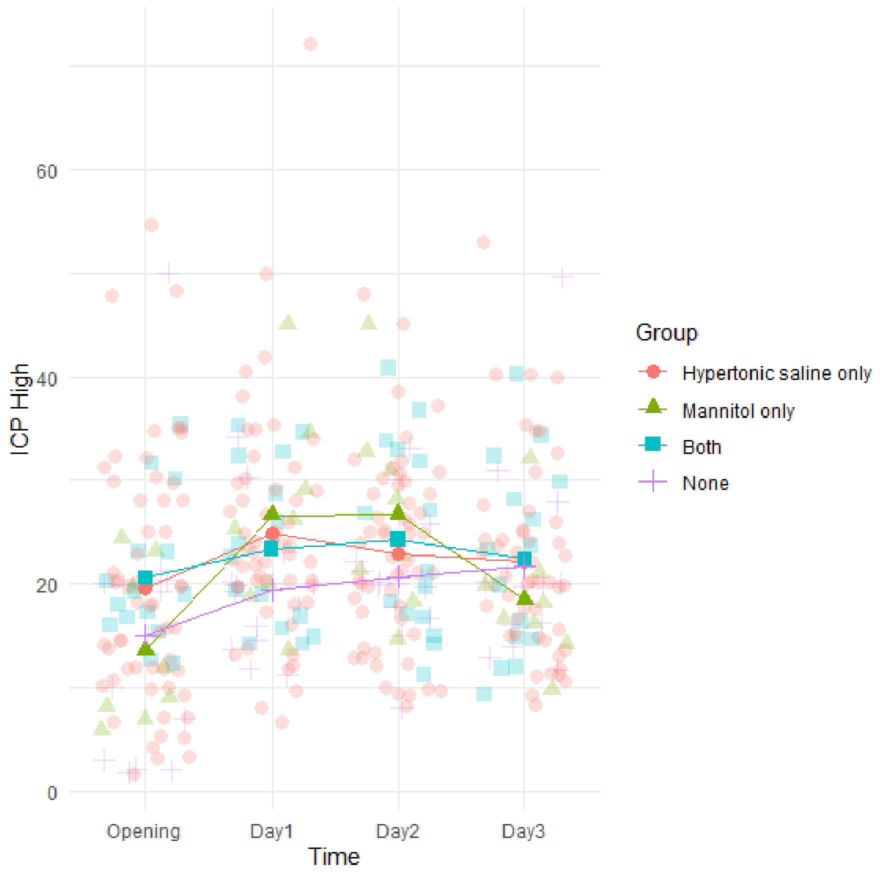

© 2025 Chong SL et al. *JAMA Network Open*.

eFigure 4. Individual and Mean of Highest Cerebral Perfusion Pressure (CPP) Readings by Day

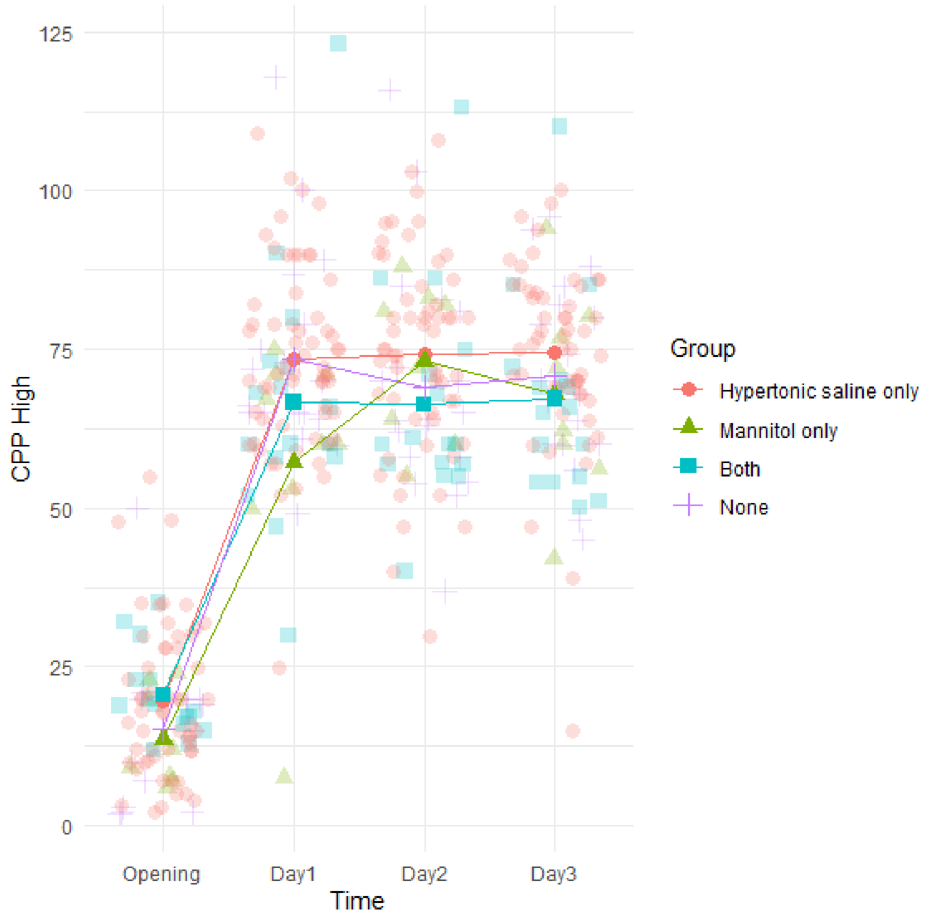

© 2025 Chong SL et al. *JAMA Network Open*.

eFigure 5. Individual and Mean of Lowest Cerebral Perfusion Pressure (CPP) Readings by Day

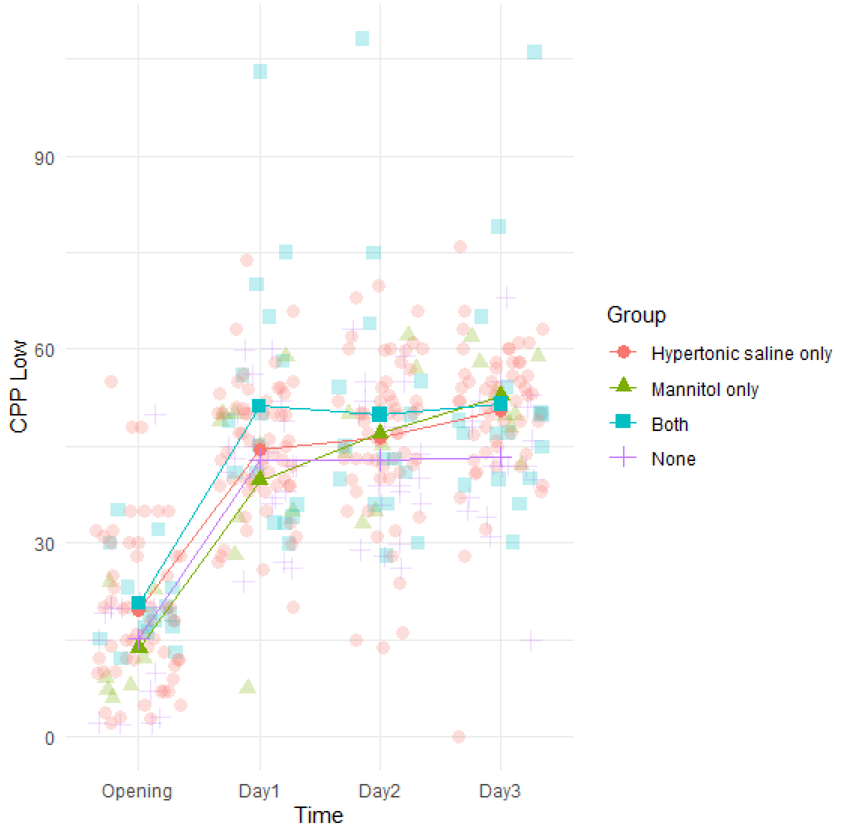

© 2025 Chong SL et al. *JAMA Network Open*.

eFigure 6. Distribution of Dosages of Hypertonic Saline (HTS) Bolus Loading Dose (mL/kg)

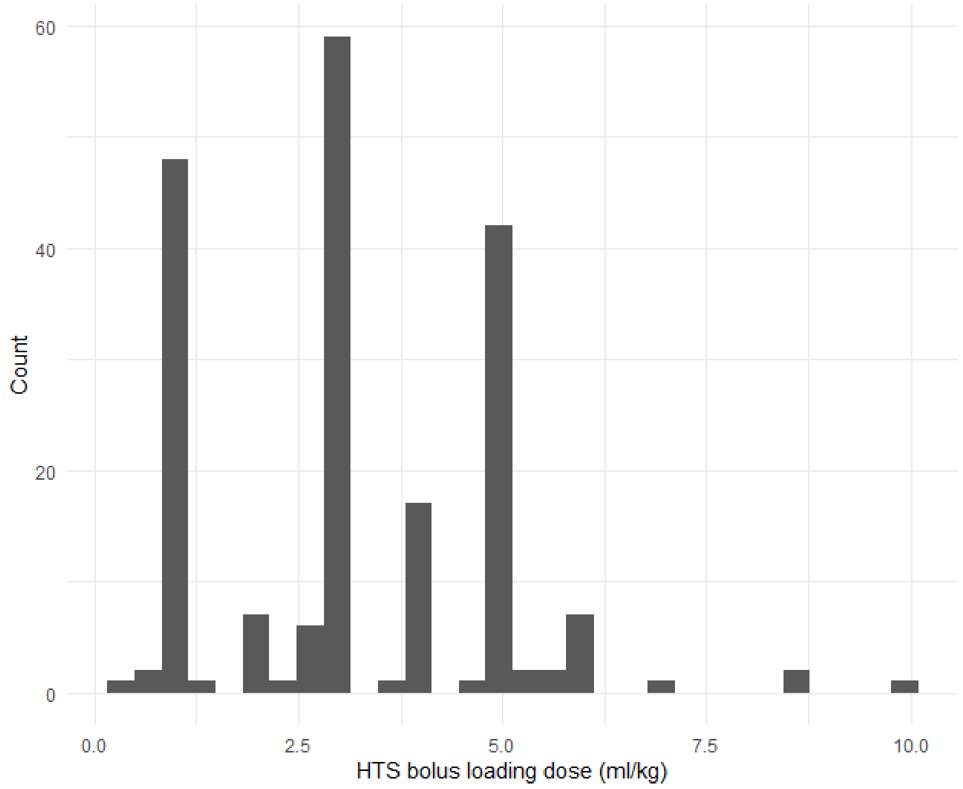

© 2025 Chong SL et al. *JAMA Network Open*.

eFigure 7. Distribution of Dosages of Hypertonic Saline (HTS) Infusion (mL/kg/h)

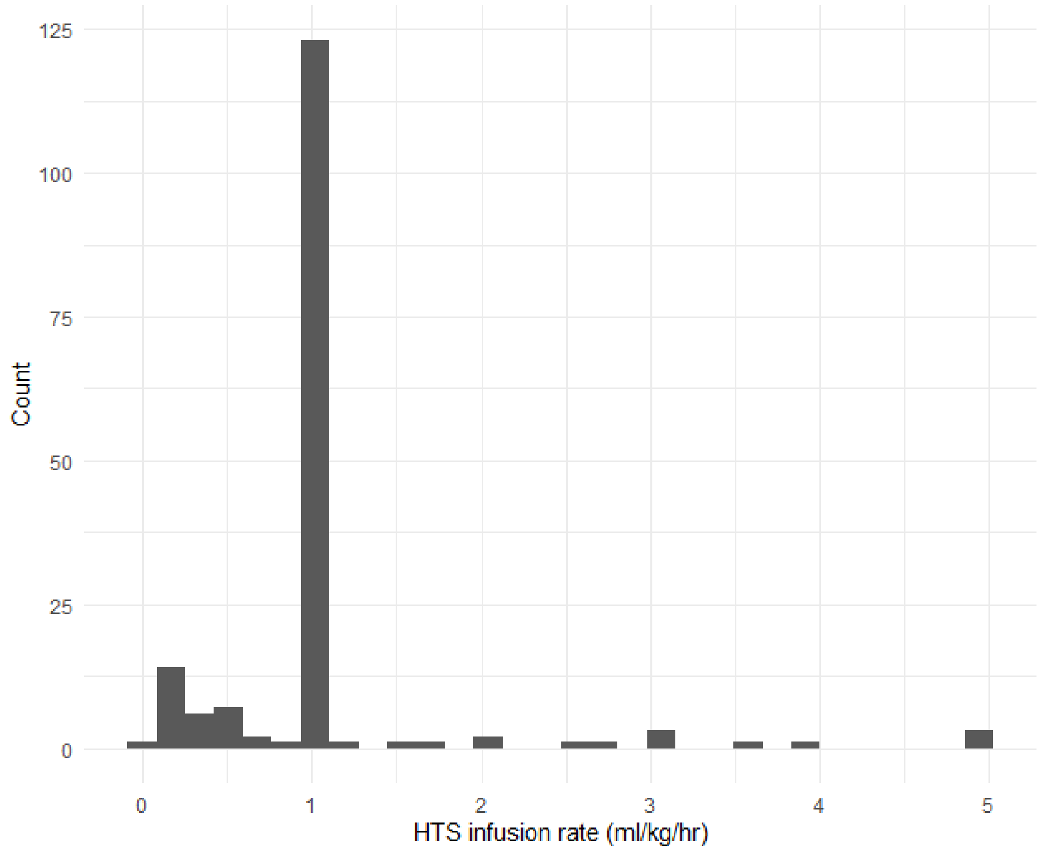

© 2025 Chong SL et al. *JAMA Network Open*.

eFigure 8. Distribution of Dosages of Mannitol Bolus Loading Dose (mL/kg)

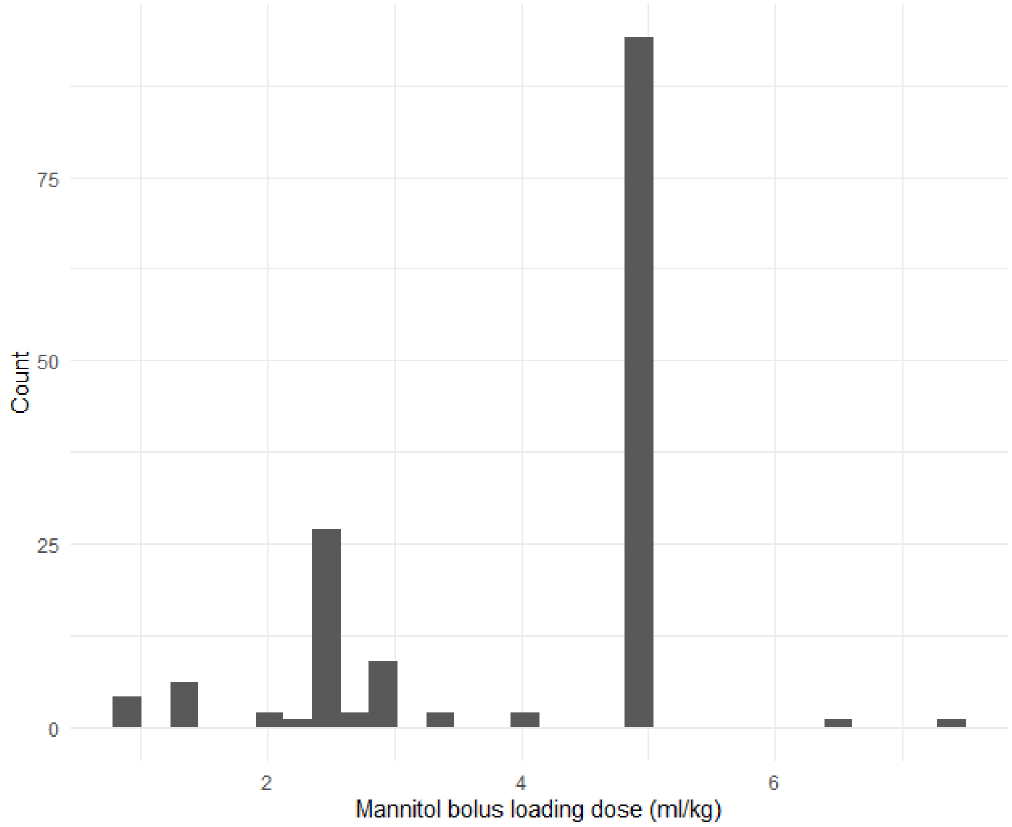

© 2025 Chong SL et al. *JAMA Network Open*.
