# Supplementary material for: Clinical Outcomes of Hypertonic Saline vs Mannitol Treatment Among Children With Traumatic Brain Injury
Source: JAMA Netw Open. 2025 Mar 11;8(3):e250438. doi: 10.1001/jamanetworkopen.2025.0438 (PMC11897838; doi:10.1001/jamanetworkopen.2025.0438)
Supplement: Supplement 2. — Nonauthor Collaborators. Pediatric Acute and Critical Care Medicine in Asia Network and Red Colaborativa Pediátrica de Latinoamérica [file jamanetwopen-e250438-s002.pdf]

Supplement 2. Nonauthor Collaborators
*First name, last name, and suffix (if applicable) are required and will appear in PubMed.

*Group Name(s): Pediatric Acute & Critical Care Medicine in Asia Network (PACCMAN) and Red Colaborativa Pediatrica de Latinoamerica (LARed)							
***First Name and Middle Initial(s)**	***Last Name**	***Suffix (eg, Jr, III)**	Academic Degrees	Institution	Location (city, state/province, country)	Role or Contribution, eg, chair, principal investigator	Group (if more than 1 Group listed in the byline) and/or Subgroup (eg, Steering Committee)
Yasser M.	Kazzaz		MBBS, FRCPC, MPH	KING ABDULLAH INTERNATIONAL MEDICAL RESEARCH CENTER	Riyadh, Saudi Arabia		PACCMAN
Ji	Jian		MD	Beijing Children's Hospital, Capital Medical University	Beijing, China		PACCMAN
Suyun	Qian		MD	Beijing Children's Hospital, Capital Medical University	Beijing, China		PACCMAN
Lijia	Fan		MBBS, MMed (Paeds), MRCPCH (UK), MCI	National University Hospital	Singapore, Singapore		PACCMAN
Olive Pei Ee	Lee		mrcpch (UK)	Department of Paediatrics, Sarawak General Hospital	Sarawak, Malaysia		PACCMAN
Soo Lin	Chuah		MD, MRCPCH (UK)	Department of Paediatrics,	Kuala Lumpur, Malaysia		PACCMAN
Kai	You		MD	Pediatric department of Shengjing	Shenyang, Liaoning, China		PACCMAN
Tao	Zhang		PhD	Pediatric department of Shengjing hospital, China Medical University	Shenyang, Liaoning, China		PACCMAN
Deiby Lasso	Noguera		MD	Hospital Infantil Los Angeles	Pasto, Colombia		LARed
Esteban	Cerón		MD	Hospital Infantil Los Angeles	Pasto, Colombia		LARed
Andrea Leal	Barceló		PhD	Hospital Universitario Virgen de la Arrixaca	Murcia, Spain		LARed
Susana Beatriz Reyes	Domínguez		MD	Hospital Universitario Virgen de la Arrixaca	Murcia, Spain		LARed
Cesia J Ortega	Ocas		MD	Hospital Nacional Hipólito Unanue	Lima, Peru		LARed
