# Supplementary material for: Clinical Outcomes of Hypertonic Saline vs Mannitol Treatment Among Children With Traumatic Brain Injury
Source: JAMA Netw Open. 2025 Mar 11;8(3):e250438. doi: 10.1001/jamanetworkopen.2025.0438 (PMC11897838; doi:10.1001/jamanetworkopen.2025.0438)
Supplement: Supplement 3. — Data Sharing Statement [file jamanetwopen-e250438-s003.pdf]

Data Sharing Statement

Chong. Clinical Outcomes of Hypertonic Saline vs Mannitol Treatment Among Children With Traumatic Brain Injury. *JAMA Netw Open*. Published March 11, 2025.
doi:10.1001/jamanetworkopen.2025.0438

Data
Data available: Yes
Data types: Deidentified participant data
How to access data: De-identified patient data can be shared upon reasonable request, made to the Corresponding Author
When available: With publication

Supporting Documents
Document types: None

Additional Information
Who can access the data: Researchers whose proposed use of the data has been approved.
Types of analyses: For medical research purposes on Traumatic brain injury research
Mechanisms of data availability: With signed data access agreement
